# A Multi-Task Grasp Detection Network Based on Global Channel–Spatial Attention Mechanism for Industrial Workshop Environments

**DOI:** 10.3390/s26165257

**Published:** 2026-08-19

**Authors:** Shengze Zhang, Zhaochun Li, Yucheng Wang, Shuyou Wang

**Affiliations:** 1 College of Mechanical and Electronic Engineering, Nanjing Forestry University, Nanjing 210037, China; zhangsz@njfu.edu.cn (S.Z.); wsycumtqsy@njfu.edu.cn (S.W.); 2 Institute of Intelligent Machines, Hefei Institutes of Physical Science, Chinese Academy of Sciences, Hefei 230031, China

**Keywords:** robotic manipulation, multi-task learning, workshop object perception, feature reconstruction, eye-in-hand calibration, RGB-D 3D localization

## Abstract

To improve the accuracy and robustness of target grasp detection and classification for robotic manipulators in unstructured environments such as workshops, a multi-task grasp detection algorithm based on a global channel–spatial attention mechanism is proposed. Through the global channel attention module, the network is able to extract richer semantic information while reducing redundant feature information, thereby improving overall performance. The effectiveness of the proposed algorithm is validated on the Cornell dataset and a self-built dataset. The grasp prediction accuracy reaches 99.5% on the Cornell dataset, while on the self-built dataset the grasp detection accuracy reaches 98.5% and the object classification accuracy reaches 99.5%. To further verify the effectiveness of the algorithm, grasping experiments are conducted on an AUBO-i3 robotic manipulator. The results show that the average grasping accuracy reaches 95% for unseen daily items and common workshop objects, which is 8.75% higher than the baseline GGCNN network. These results indicate that the proposed algorithm can effectively perform grasping tasks for robotic manipulators in unstructured environments.

## 1. Introduction

Robotic manipulation has become an important enabling technology for automated manufacturing, assembly, sorting, and material handling. A fundamental requirement for autonomous manipulation is the ability to identify a feasible grasp configuration from visual observations and convert the perception result into an executable robot action. Compared with structured production lines in which object positions and orientations are predetermined, workshop environments present greater perceptual challenges because objects may exhibit considerable variations in shape, scale, orientation, illumination, and background appearance. Consequently, reliable and efficient visual grasp perception remains an important problem for deploying robotic manipulators in practical workshop scenarios [1,2,3].

Among vision-based robotic grasping approaches, planar grasp detection provides an efficient representation for parallel-jaw manipulation by describing a grasp using its image-plane position, orientation, and gripper opening. Deep learning-based approaches have substantially improved the accuracy and inference efficiency of planar grasp detection by directly learning grasp-related visual features from images [1,2,4]. Nevertheless, two challenges remain particularly relevant to workshop-oriented manipulation. First, grasp detection requires both semantic discrimination and spatially precise localization. Deep features with strong semantic responses are useful for distinguishing objects from complex backgrounds, whereas accurate grasp prediction requires preserving local geometric and spatial information associated with feasible contact regions. Conventional convolutional feature extraction and individual channel or spatial attention operations may not always provide sufficient interaction between these complementary feature dimensions. Second, redundant spatial and channel responses can propagate into the grasping head during multi-scale feature fusion, potentially weakening grasp-relevant representations and introducing unnecessary computational overhead.

In addition to grasp pose estimation, practical sorting and manipulation systems often require information about the category of the manipulated object. A conventional solution is to perform object recognition and grasp detection using separate perception models. Although such a pipeline provides task-specific predictions, it introduces duplicated feature extraction and separates two perception tasks that operate on the same visual observation. A shared multi-task architecture provides an alternative by extracting common visual representations and producing object category and grasp pose predictions within a unified network [5,6,7]. However, the two tasks do not necessarily benefit each other equally: category recognition emphasizes semantic invariance, whereas grasp detection depends more strongly on spatially localized geometric information. Therefore, an appropriate multi-task grasping framework should preserve task-specific information while sharing common representations rather than assuming that an auxiliary classification objective will necessarily improve grasp detection accuracy.

Recent attention mechanisms provide effective means of enhancing feature representation by adaptively recalibrating feature responses [8,9,10,11]. Channel attention approaches emphasize informative semantic channels, while spatial attention mechanisms highlight discriminative regions in the feature map. Sequential channel–spatial attention further combines these two dimensions. Nevertheless, simply applying the two attention operations does not explicitly encourage information exchange among channel groups before spatial refinement. For grasp perception, such interaction may be useful because graspable regions are determined by both semantic channel responses and their spatial distribution. Motivated by this observation, we introduce a Global Channel–Spatial Attention (GCSA) module that sequentially performs channel feature recalibration, channel shuffling, and spatial feature refinement. The channel shuffling operation is inserted between the channel and spatial attentions to promote cross-group feature interaction before spatial weighting. While channel–spatial attention itself is not a new concept, the proposed design focuses on how these operations are organized within the hierarchical backbone for grasp-oriented feature extraction.

Another consideration is the representation redundancy introduced during hierarchical feature fusion. Multi-scale features are important for grasp detection because high-level features provide semantic information while shallow features preserve spatial details. However, directly aggregating these features may also retain redundant spatial responses and correlated channel representations. Inspired by spatial and channel reconstruction mechanisms [12], we develop a Spatial and Channel Reconstruction Grasping Module (SCRGM) for task-specific grasp prediction. The module integrates spatial reconstruction and channel reconstruction with progressive feature upsampling and grasp map prediction, aiming to suppress redundant responses while preserving information relevant to grasp localization.

### 1.1. Related Work

#### 1.1.1. Planar Robotic Grasp Detection

Vision-based robotic grasp detection aims to determine feasible grasp configurations from visual observations and provide executable pose information for robotic manipulators. According to the representation space of the grasp pose, existing approaches can generally be categorized into planar grasp detection and six-degrees-of-freedom (6-DoF) grasp detection, with 6-DoF grasping methods typically relying on depth images or point clouds to estimate grasp positions and end-effector orientations in three-dimensional space. Large-scale benchmarks and representative point-cloud approaches, including GraspNet-1Billion, Contact-GraspNet, GSNet, and AnyGrasp, have substantially advanced general-object grasp perception in cluttered scenes [13,14,15,16]. Although these methods provide greater flexibility for spatial manipulation, they generally require reliable 3D geometric information and involve relatively complex procedures for point-cloud feature extraction, grasp candidate generation, and pose estimation. In contrast, planar grasp detection represents a grasp using its center position, orientation, and gripper opening in the image plane. This representation is particularly suitable for parallel-jaw manipulation in tabletop and workshop environments thanks to its relatively simple formulation and computational efficiency [1,4].

Early deep learning-based planar grasping approaches mainly formulated grasp detection as a grasp rectangle regression problem. Redmon et al. transformed sliding window-based grasp detection into an end-to-end regression problem, allowing a neural network to directly predict grasp rectangles from visual observations [17]. This work demonstrated the potential of deep learning for robotic grasp detection, although directly regressing a limited number of grasp candidates may be less effective when an object has multiple feasible grasp regions. Morrison et al. subsequently proposed GG-CNN, which formulated grasp detection as a dense pixel-wise prediction problem and simultaneously generated grasp quality, grasp angle, and gripper width maps [18]. Its compact fully convolutional architecture enabled efficient inference and provided an important basis for real-time closed-loop robotic grasping; however, its relatively lightweight feature extractor may have limited representation capability under complex backgrounds, occlusion, and substantial appearance variations.

Subsequent studies have introduced residual learning, multi-scale feature fusion, and alternative grasp representations to improve detection performance. GR-ConvNet and its variants employ residual convolutional structures to generate dense grasp quality, angle, and width maps, and have demonstrated favorable performance on benchmark datasets such as Cornell and Jacquard [19]. Keypoint-based approaches represent a grasp using paired keypoints or other geometric primitives, thereby reducing some of the difficulties associated with directly regressing rotated grasp rectangles [20]. Other studies have explored lightweight encoder–decoder architectures, multi-scale feature aggregation, and efficient feature enhancement mechanisms to achieve a better balance between grasp detection accuracy and real-time inference efficiency [21,22]. Keypoint-based FANet further combines local and global feature refinement with grasp keypoint optimization for accurate real-time detection [23]. Nie et al. investigated a compact RGB-D grasp detector based on unequal feature encoding and knowledge distillation, emphasizing deployment-oriented trade-offs between model size and accuracy [24].

Recent studies have continued to investigate the combination of convolution, attention, hierarchical feature extraction, and lightweight multi-scale design. UFGNet employs a hierarchical transformer encoder, convolution–self-attention feature aggregation, residual decoding, and Shuffle Attention, reporting a Cornell accuracy of 98.4% [25]. LPGNet adopts a MobileNet-based encoder–decoder and a lightweight multi-scale feature enhancement module; the published Cornell results are 98.31% under the image-wise split and 97.84% under the object-wise split, with a reported inference time of 14.2 ms [26]. These recent methods further demonstrate the importance of balancing local feature extraction, contextual modeling, multi-scale representation, and real-time inference in planar grasp detection.

More recently, transformer-based architectures and their variants have been introduced into robotic grasp perception to improve contextual feature representation and long-range dependency modeling [27,28]. Hierarchical Vision Transformer and MetaFormer-style architectures can extract semantic information at multiple spatial resolutions, making them potentially suitable for multi-scale grasp prediction. Nevertheless, conventional self-attention can introduce considerable computational overhead as the spatial resolution increases, which may restrict its application in real-time robotic systems. Therefore, improving semantic and spatial feature interaction without substantially increasing computational complexity remains an important consideration for efficient planar grasp detection.

Beyond benchmark accuracy, recent 6-DoF research has increasingly emphasized generalization to novel objects and viewpoints. Physical domain priors have been incorporated to regularize grasp prediction on objects with diverse structures [29]; NeuGraspNet uses neural surface rendering for any-view grasp prediction in clutter [30], while ZeroGrasp jointly models shape reconstruction and grasp pose prediction to improve zero-shot transfer [31]. Although these methods address a different grasp representation from the planar formulation studied here, they highlight generalization, geometric consistency, and deployment on previously unseen objects as important directions for robotic grasp perception.

Most existing planar grasp detection networks primarily focus on estimating grasp configurations, such as grasp quality, orientation, and gripper width. In practical sorting and manipulation applications, however, the robot may also need to determine the semantic category of the manipulated object. Employing independent recognition and grasp detection networks results in duplicated feature extraction and increases the complexity of the perception pipeline. This motivates the development of unified architectures capable of jointly exploiting semantic and geometric information for object recognition and grasp prediction.

#### 1.1.2. Multi-Task Robotic Grasp Perception

Multi-task learning enables multiple related tasks to share feature representations within a unified network, potentially reducing redundant computation while exploiting complementary information across tasks [32]. In robotic grasping, previous studies have combined grasp detection with semantic segmentation, instance segmentation, object-relation reasoning, and language understanding. Duan et al. developed a semantic robotic grasping framework for stacking scenes that jointly exploits semantic information and grasp prediction within a multi-task architecture [5]. Attribute-based robotic grasping has also combined visual observations with semantic attribute descriptions and data-efficient adaptation, demonstrating another route for coupling recognition cues with grasp-affordance prediction [33].

In approaches combining semantic segmentation with grasp detection, the segmentation task can provide object boundaries and semantic regions that facilitate object-specific grasp prediction. For example, multi-task grasping architectures have jointly performed semantic segmentation and grasp detection so that predicted grasp candidates can be associated with individual objects in a scene [34]. Instance-level approaches further integrate instance segmentation with grasp prediction to distinguish graspable regions belonging to different objects in cluttered environments [6,35]. Other studies have exploited segmentation-derived boundary information to improve local feature representation around contact regions, particularly when objects are partially occluded or closely arranged [34]. Although these approaches provide richer spatial priors for grasp detection, they generally require pixel-level segmentation annotations, which increases the cost of dataset construction.

Beyond segmentation, multi-task robotic grasping has also incorporated manipulation relationships and grasp-order reasoning. In cluttered or stacked scenes, selecting a geometrically feasible grasp without considering surrounding objects may lead to collisions or unsuccessful manipulation. Some methods jointly estimate grasp configurations and object relationships to determine appropriate manipulation sequences [36,37]. Target-oriented grasping approaches additionally incorporate occlusion reasoning, shape completion, or target-conditioned information to identify a specified object and generate its corresponding grasp pose [37,38]. These studies extend grasp perception from purely geometric prediction towards integrated scene understanding and manipulation reasoning.

With the development of multimodal robotic perception, language and tactile information have increasingly been incorporated into robotic manipulation systems. Language-conditioned grasping methods use textual instructions to identify target objects and generate corresponding manipulation actions, enabling joint reasoning over visual observations, language instructions, and manipulation objectives [39]. Visual–tactile approaches exploit contact, pressure, deformation, or slip information from tactile sensors to refine visually estimated grasp configurations and improve grasp stability [40,41]. Beyond grasping, Mao et al. integrated a six-bar tensegrity structure with 24 flexible sensors and a fine-tuned multimodal large language model to support self-shape recognition, state monitoring, and fault diagnosis [42]. This recent study illustrates a broader trend toward combining flexible sensing with multimodal reasoning for autonomous state cognition. These developments demonstrate the potential of multimodal perception for intelligent robotic systems, although they generally require additional sensing hardware, multimodal annotations, or more complex inference and control pipelines.

In contrast to these approaches, the present study focuses specifically on the joint prediction of object categories and planar grasp configurations for workshop tools and parts. The proposed framework does not require pixel-level segmentation masks, language instructions, or tactile measurements as network inputs; instead, hierarchical RGB features are shared between an object classification branch and a grasp detection branch. Importantly, these two tasks do not necessarily require identical feature representations: classification favors semantically discriminative and relatively invariant representations, whereas grasp detection depends more strongly on spatially localized geometric information. Consequently, feature sharing may provide computational benefits, but can also introduce competition between task objectives, such as conflicting or imbalanced task gradients [32,43]. For this reason, in this study we do not assume that the auxiliary classification task necessarily improves grasp accuracy; instead, the relationship between the two tasks is evaluated through single-task/multi-task comparisons and classification loss weight sensitivity experiments.

#### 1.1.3. Attention Mechanisms and Feature Reconstruction

Attention mechanisms have been widely adopted to adaptively recalibrate feature responses and emphasize task-relevant information. According to the dimension on which attention is applied, visual attention mechanisms can generally be divided into channel attention, spatial attention, and joint channel–spatial attention.

Channel attention mechanisms assign adaptive weights to individual feature channels according to their semantic importance. The Squeeze-and-Excitation (SE) mechanism uses global information aggregation followed by channel-wise transformation to model dependencies among feature channels [8]. Efficient Channel Attention (ECA) subsequently introduces lightweight local cross-channel interaction to reduce the additional parameter overhead associated with channel recalibration [9]. Such approaches can strengthen semantically informative channels but generally apply the learned channel weights across all spatial locations, providing limited explicit modeling of spatially localized grasp regions.

In contrast, spatial attention mechanisms learn spatial weighting maps in order to highlight informative locations within feature maps. For robotic grasp detection, spatial attention can potentially emphasize object boundaries, candidate contact regions, and areas around feasible grasp centers while suppressing irrelevant background responses. However, spatial weighting alone provides limited modeling of dependencies among semantic feature channels. To address this issue, joint channel–spatial attention mechanisms have been developed. For example, CBAM sequentially applies channel and spatial attention to refine features along both dimensions [10], while Shuffle Attention incorporates grouped feature processing and channel shuffling to facilitate interaction between information from different feature groups [11]. These methods provide important foundations for jointly exploiting channel and spatial information.

The proposed GCSA module is also based on the principle of joint channel and spatial feature enhancement. Therefore, the novelty of GCSA does not lie in the use of channel and spatial attention within the same module; instead, GCSA proposes organizing the feature enhancement process as follows: channel recalibration → channel shuffling → spatial refinement.

Compared with channel-oriented mechanisms such as SE and ECA, GCSA additionally performs spatial refinement to emphasize grasp-relevant regions. Compared with sequential channel–spatial mechanisms such as CBAM, channel shuffling is inserted between channel recalibration and spatial refinement to facilitate cross-group feature interaction before spatial weighting. The resulting module is incorporated as a feature mixing operation within hierarchical GCSAFormer blocks. To determine whether this organization provides meaningful benefits rather than merely increasing model complexity, the revised experimental evaluation includes comparisons with representative attention mechanisms along with component-level ablation studies.

In addition to attention-based feature enhancement, reducing redundant spatial and channel responses represents another direction for improving feature representation and computational efficiency. Conventional convolution operations may preserve correlated or low-information feature responses across spatial locations and channels. For example, Spatial and Channel Reconstruction Convolution (SCConv) introduces a Spatial Reconstruction Unit (SRU) and Channel Reconstruction Unit (CRU) to respectively reduce spatial and channel redundancy [12]. The SRU separates and reconstructs spatial features according to their information content, whereas the CRU employs channel splitting, compression, grouped transformation, and adaptive fusion to improve channel representation [12]. These reconstruction operations provide an efficient means of reducing feature redundancy, although they were originally proposed as general-purpose convolutional feature processing mechanisms rather than task-specific grasp prediction modules.

Inspired by these spatial and channel reconstruction mechanisms, this study integrates SRU and CRU into a task-specific grasping head and combines them with multi-scale feature aggregation, progressive upsampling, and grasp map prediction to construct the Spatial and Channel Reconstruction Grasping Module (SCRGM). We do not claim SRU or CRU themselves as newly introduced fundamental operations; instead, the contribution lies in their task-oriented integration into the grasp prediction stage in order to refine fused features before estimating grasp quality, orientation, and gripper width. The individual effects of SRU and CRU are further investigated through dedicated ablation experiments.

In summary, while substantial progress has been achieved in planar grasp detection, multi-task robotic perception, channel–spatial attention, and feature reconstruction, current workshop-oriented robotic manipulation still requires an efficient means of jointly predicting object semantics and spatially precise grasp configurations while controlling the computational overhead associated with feature enhancement. Motivated by these considerations, the proposed framework introduces GCSA into a hierarchical backbone to enhance channel–spatial feature interaction and incorporates SCRGM into the grasping head to refine redundant multi-scale features, forming a unified multi-task grasp perception architecture.

### 1.2. Objectives and Contributions

Based on these considerations, this study aims to develop an efficient RGB-based multi-task grasp perception framework for workshop-oriented robotic manipulation. The proposed MTGCSAFormer uses a hierarchical GCSAFormer backbone to extract multi-scale visual features, a feature fusion mechanism to aggregate semantic and spatial information, an auxiliary classification branch to identify object categories, and an SCRGM-based grasping head to predict planar grasp configurations. The network itself uses RGB images for visual inference. During physical robot execution, the predicted two-dimensional grasp location is subsequently associated with depth information for three-dimensional localization and transformed into the robot coordinate system through camera–robot calibration [44,45]. This distinction allows the perception network to remain RGB-based while enabling executable grasping actions in three-dimensional space.

The main contributions of this work are summarized as follows:

1. A unified multi-task framework for object recognition and planar grasp detection is developed. MTGCSAFormer shares hierarchical visual features between the two tasks and simultaneously predicts object categories and grasp configurations. The multi-task formulation is intended to provide integrated perception and shared computation rather than assuming that the classification task necessarily improves grasp detection accuracy.
2. A GCSA-based hierarchical backbone is introduced for grasp-oriented feature extraction. The proposed GCSA module organizes channel attention, channel shuffling, and spatial attention in a sequential manner, allowing for cross-group channel interaction before spatial weighting. Its individual components are evaluated through component-level ablation and comparison with representative attention mechanisms, while practical execution efficiency is reported using the measured per-image inference time.
3. A grasping head based on spatial and channel reconstruction is developed for multi-scale grasp prediction. Inspired by existing spatial and channel reconstruction mechanisms, SCRGM integrates spatial reconstruction, channel reconstruction, progressive upsampling, and task-specific grasp map prediction to reduce redundant feature responses. The proposed framework is evaluated on the Cornell Grasping Dataset and the workshop-oriented GTPWD, followed by physical grasping experiments using a robotic manipulator.

The remainder of this paper is organized as follows: Section 2 presents the proposed MTGCSAFormer architecture, including the GCSAFormer backbone, multi-scale feature fusion, SCRGM grasping head, multi-task prediction, and robot coordinate transformation; Section 3 provides the datasets, evaluation protocols, implementation details, comparative experiments, ablation studies, input resolution analysis, physical grasping experiments, and a discussion of the principal findings and limitations; finally, Section 4 concludes the paper and outlines future research directions.

## 2. Proposed Method

### 2.1. System Overview and Task Definition

**Figure 1:**
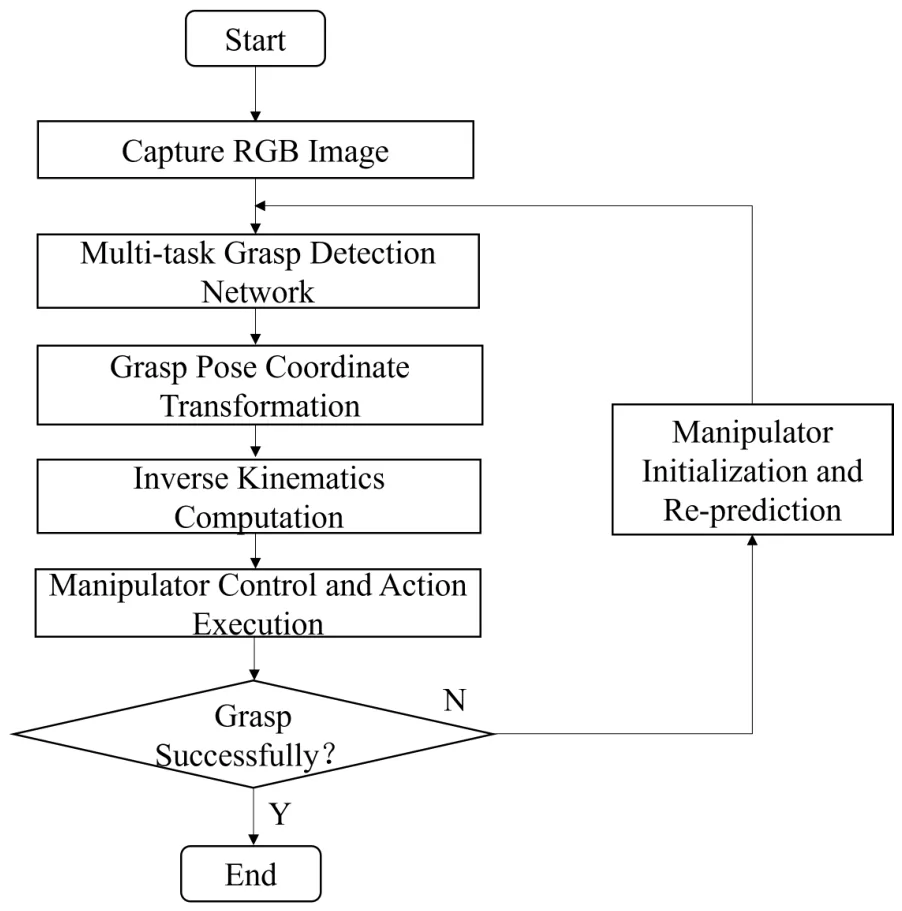
Complete robotic grasping pipeline. MTGCSAFormer predicts an object category and a two-dimensional grasp from an RGB image, while depth association and camera-to-robot transformation are subsequently used for physical execution. The highlighted MTGCSAFormer perception block is detailed in Figure 2.

**Figure 2:**
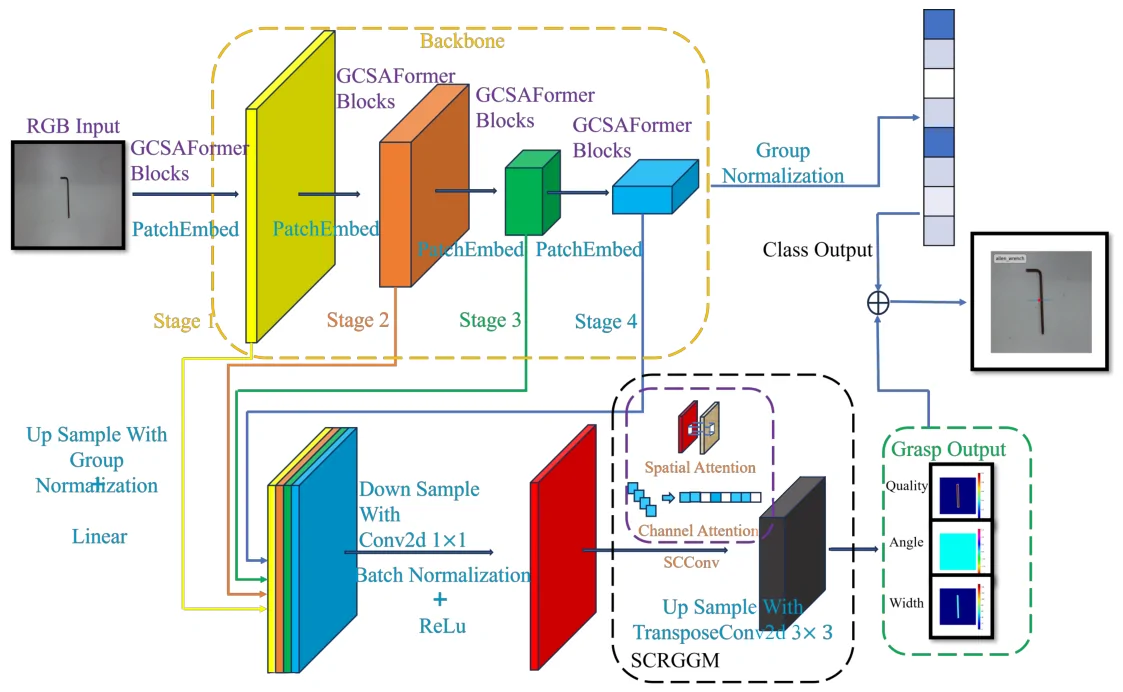
Detailed architecture of the MTGCSAFormer perception block highlighted in Figure 1. The hierarchical GCSAFormer backbone extracts four scale features for the object classification head and the multi-scale SCRGM grasp prediction branch. The internal structures of GCSA and SCRGM are detailed in Figure 3 and Figure 4, respectively.

The complete robotic grasping pipeline is illustrated in Figure 1. The MTGCSAFormer perception block highlighted in this figure is expanded in Figure 2.

The system consists of RGB image acquisition, multi-task grasp perception, grasp pose decoding, three-dimensional localization, coordinate transformation, motion planning, and robotic execution. An RGB image of the workspace is first captured and fed into the proposed MTGCSAFormer. The network simultaneously predicts the object category and dense planar grasp maps. The predicted two-dimensional grasp is subsequently associated with depth information to recover its three-dimensional position and is transformed from the camera coordinate system to the robot base coordinate system.

It should be emphasized that MTGCSAFormer is an RGB-based perception network. Depth information is not used by the backbone, attention module, feature fusion module, grasping head, or classification branch. Instead, the aligned depth image is used after network inference to convert the predicted image-plane grasp position into an executable three-dimensional robot position. Therefore, the network input modality and the sensing information used for robot execution are clearly separated.

Given an RGB image

$$I\in R^{B\times 3\times H\times W},$$

the complete network produces an object classification output and four dense grasp maps

(1) $$\left\{O_{\mathrm{cls}},Q,C,S,W_{g}\right\}=\mathrm{MTGCSAFormer}\left(I\right),$$

where $O_{\mathrm{cls}}$ denotes the class logits for object classification, *Q* represents the grasp quality map, *C* and *S* are the orientation-encoded feature maps, and $W_{g}$ indicates the gripper width map. The notation $W_{g}$ is adopted to distinguish the predicted gripper opening width from the image width *W* and avoid symbol ambiguity.

The dense network outputs are further decoded into a standard planar grasp representation

(2) $$g_{2D}=\left(u,v,\theta ,w_{g}\right),$$

where (u,v) is the grasp center in the image coordinate system, θ denotes the in-plane grasp orientation of the parallel gripper, and $w_{g}$ corresponds to the required gripper opening width for grasping.

**Figure 3:**
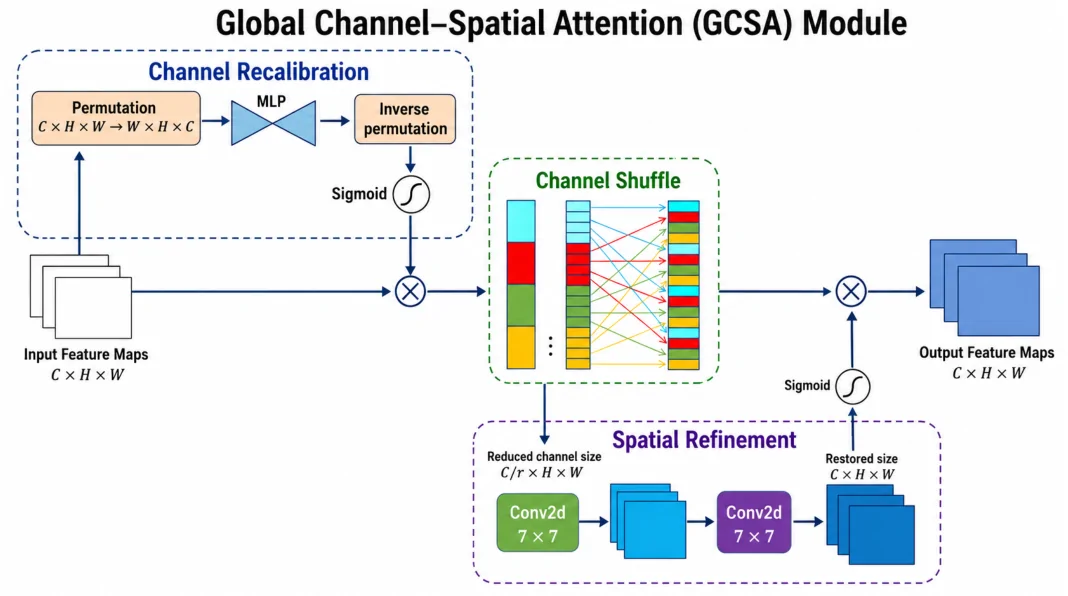
Detailed structure of the GCSA module employed in the GCSAFormer backbone shown in Figure 2. GCSA sequentially performs channel attention, channel shuffling, and spatial attention.

**Figure 4:**
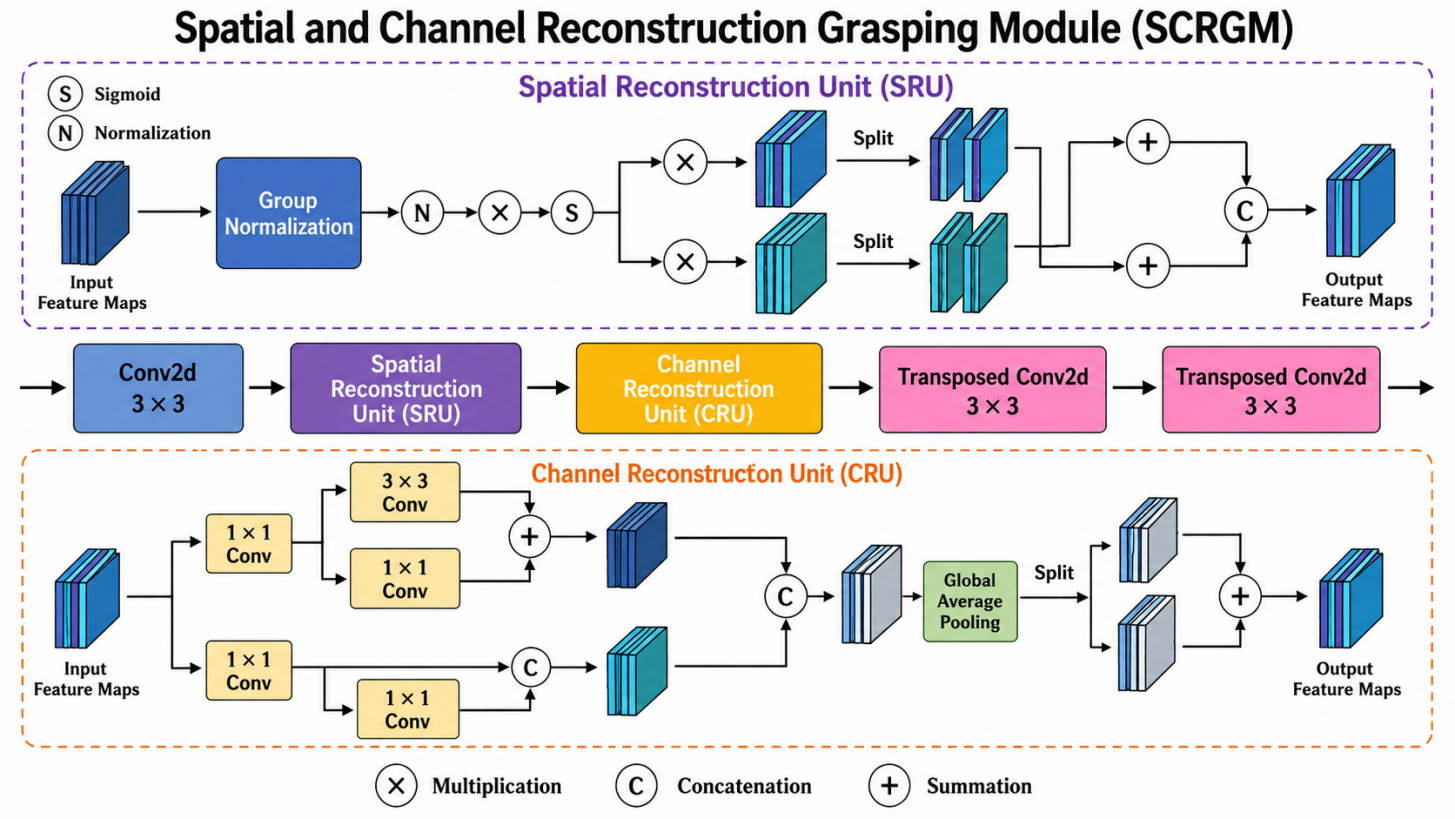
Detailed architecture of the Spatial and Channel Reconstruction Grasping Module (SCRGM) used in the grasp prediction branch of Figure 2. The fused representation is processed sequentially by the Spatial Reconstruction Unit (SRU) and Channel Reconstruction Unit (CRU), followed by two transposed convolution layers for spatial resolution recovery.

### 2.2. Overall Architecture of MTGCSAFormer

The internal architecture of MTGCSAFormer is shown in Figure 2. In contrast to Figure 1, which presents the complete robotic system, Figure 2 provides an enlarged view of the highlighted multi-task perception block. The proposed network consists of four principal components: a hierarchical GCSAFormer backbone, a multi-scale feature fusion module, an auxiliary classification head, and a reconstruction-based grasping head.

The architecture is motivated by the different feature requirements of object classification and planar grasp detection. Classification mainly relies on high-level semantic representations, whereas grasp detection additionally requires precise spatial information for locating grasp centers, estimating grasp orientations, and predicting gripper openings. The proposed architecture extracts hierarchical features at different resolutions and uses task-specific heads after shared feature extraction.

The GCSAFormer backbone generates four hierarchical feature maps

(3) $$\left\{F_{1},F_{2},F_{3},F_{4}\right\}=B_{\mathrm{GCSA}}\left(I\right),$$

where $F_{1}$ and $F_{2}$ retain detailed spatial information while $F_{3}$ and $F_{4}$ encode stronger semantic representations. These multi-scale hierarchical features are aligned and aggregated through the fusion module

(4) $$F_{\mathrm{fuse}}=F\left(F_{1},F_{2},F_{3},F_{4}\right),$$

where F(·) denotes the overall operations of channel alignment, upsampling, and feature concatenation. The fused feature is subsequently fed into the grasping head for dense grasp prediction:

(5) $$\left\{Q,C,S,W_{g}\right\}=H_{\mathrm{grasp}}\left(F_{\mathrm{fuse}}\right)$$

while the deepest high-semantic feature is utilized for object category classification:

(6) $$O_{\mathrm{cls}}=H_{\mathrm{cls}}\left(F_{4}\right).$$

The classification and grasping branches share the hierarchical GCSAFormer backbone but adopt independent task-specific prediction heads. This design avoids redundant feature extraction and enables unified object recognition and planar grasp prediction within a single network. This study does not assume that the auxiliary classification task necessarily improves grasp detection performance. Object classification emphasizes semantic invariance, while grasp detection relies heavily on spatially localized geometric details. The actual influence of the classification branch is quantitatively evaluated through single-task and multi-task comparative experiments as well as loss-weight sensitivity analyses, which are presented in Section 4.

### 2.3. GCSAFormer Backbone

In this work, the backbone adopts the GCSAFormer-S24 configuration. The four stages contain 4, 4, 12, and 4 GCSAFormer blocks, respectively, yielding a total of 24 blocks. Their corresponding channel dimensions are 64, 128, 320, and 512. The input RGB image is first processed by a convolutional patch embedding layer:

(7) $$X_{0}=\mathrm{PatchEmbed}\left(I\right).$$

The patch embedding operation employs a 7×7 convolution with a stride of 4 and padding of 2, producing feature maps with a spatial resolution of approximately H/4×W/4. A 3×3 convolution with stride 2 and padding 1 is adopted for downsampling between adjacent stages. Consequently, the spatial resolutions of the four stage outputs are $\frac{H}{4}\times \frac{W}{4}$, $\frac{H}{8}\times \frac{W}{8}$, $\frac{H}{16}\times \frac{W}{16}$, and $\frac{H}{32}\times \frac{W}{32}$, respectively. Each GCSAFormer block consists of a GCSA feature mixer and a convolutional MLP. Group normalization with a single group is adopted for feature normalization. The MLP is constructed by two 1×1 convolutions with GELU activation, implementing a channel transformation of C→4C→C. The forward propagation of a single GCSAFormer block is formulated as follows:

(8) $$Y=X+\mathrm{DropPath}\left[\gamma_{1}⊙\mathrm{GCSA}\left({\mathrm{Norm}}_{1}\left(X\right)\right)\right]$$

(9) $$Z=Y+\mathrm{DropPath}\left[\gamma_{2}⊙\mathrm{MLP}\left({\mathrm{Norm}}_{2}\left(Y\right)\right)\right]$$

where $\gamma_{1}$ and $\gamma_{2}$ denote learnable layer-scale parameters. For the GCSAFormer-S24 configuration, their initial values are set to $10^{-5}$. The specific drop-path rate is provided in the implementation details according to the final training setup. When the pretrained PoolFormer-S24 checkpoint is utilized, the compatible backbone parameters are partially initialized from PoolFormer, while the parameters of the newly introduced GCSA modules are independently initialized.

### 2.4. Global Channel–Spatial Attention Module

Accurate planar grasp detection requires the network to identify semantically informative object features while preserving precise spatial responses associated with grasp centers, contact regions, and gripper orientations. Channel attention is effective for emphasizing discriminative semantic channels, whereas spatial attention highlights informative locations in the feature map. Existing joint channel–spatial attention mechanisms have demonstrated that these two types of information are complementary. However, directly applying channel attention followed by spatial attention does not explicitly encourage information exchange among channel groups before the spatial weighting operation. To address this issue, we design a Global Channel–Spatial Attention (GCSA) module. This module serves as the feature mixer within the hierarchical GCSAFormer backbone shown in Figure 2, and its internal structure is illustrated in Figure 3. The proposed module sequentially consists of a channel attention branch, a channel shuffle operation, and a spatial attention branch:

ChannelAttention→ChannelShuffle→SpatialAttention

The channel attention branch recalibrates semantically informative channel responses. The channel shuffle operation then redistributes features across channel groups to promote cross-group information interaction. Finally, the spatial attention branch assigns adaptive weights to grasp-relevant spatial regions and further refines the spatial feature representation. Therefore, the methodological contribution of GCSA does not lie in the first joint use of channel and spatial attention, but in inserting channel shuffle between the two attention branches to enhance feature interaction before spatial weighting. Given an input feature map

(10) $$X\in R^{B\times C\times H\times W}$$

where *B*, *C*, *H*, and *W* denote the batch size, number of channels, feature height, and feature width, respectively, the overall GCSA operation is formulated as

(11) $$X_{c}=X⊙M_{c}\left(X\right),$$

(12) $$X_{\mathrm{sh}}=\mathrm{Shuffle}\left(X_{c}\right),$$

(13) $$Y=X_{\mathrm{sh}}⊙M_{s}\left(X_{\mathrm{sh}}\right),$$

where $M_{c}\left(\cdot \right)$ denotes the channel attention mapping, Shuffle(·) denotes the channel shuffle operation, $M_{s}\left(\cdot \right)$ denotes the spatial attention mapping, and ⊙ represents element-wise multiplication.

#### 2.4.1. Channel Attention

The channel attention branch is designed to strengthen semantically informative channel responses while preserving the spatial arrangement of the feature map. Unlike conventional channel attention mechanisms that compress all spatial positions using global pooling before channel modeling, the proposed branch applies a two-layer channel transformation to the channel vector at each spatial location. In this way, channel dependencies are modeled without prematurely discarding spatial details that are essential for grasp localization. The channel attention map is generated as

(14) $$M_{c}\left(X\right)=\sigma \left(W_{2}\delta \left(W_{1}X\right)\right)$$

and the channel-enhanced feature is obtained by

(15) $$X_{c}=X⊙M_{c}\left(X\right),$$

where $W_{1}$ and $W_{2}$ denote learnable transformation matrices, δ(·) denotes the nonlinear activation function, and σ(·) denotes the sigmoid function. The first transformation reduces the channel dimension from *C* to C/r and the second restores it to *C*, where *r* denotes the channel reduction ratio. Through this operation, channels associated with discriminative object semantics are emphasized while less informative channel responses are suppressed, yielding semantically purified features for subsequent grasp detection.

#### 2.4.2. Channel Shuffling

After channel attention, the feature channels may still remain relatively isolated within their original groups. To promote cross-group information exchange before spatial attention, a channel shuffling operation is introduced to interleave channel features from different groups. The channel-enhanced feature is first reshaped into a grouped channel structure:

(16) $$X_{c}^{\prime }=\mathrm{Reshape}\left(X_{c},B,g,\frac{C}{g},H,W\right)$$

where *g* represents the number of channel groups. Subsequently, the group dimension and the within-group channel dimension are transposed to realize cross-group feature interaction:

(17) $$X_{c}^{\prime \prime }=\mathrm{Transpose}\left(X_{c}^{\prime },B,\frac{C}{g},g,H,W\right).$$

Finally, the rearranged feature is reshaped back to the original channel dimension:

(18) $$X_{\mathrm{sh}}=\mathrm{Reshape}\left(X_{c}^{\prime \prime },B,C,H,W\right).$$

This hierarchical reshaping and transposing operation thoroughly interleaves feature channels from different groups. Consequently, the subsequent spatial attention branch can perform spatial weighting on a fully mixed feature representation, which helps to alleviate the isolation caused by group-wise feature processing. The effectiveness and contribution of the introduced channel shuffle mechanism are quantitatively validated via ablation experiments comparing the no-shuffle baseline and the complete GCSA model.

#### 2.4.3. Spatial Attention

After cross-group channel interaction, the spatial attention branch is adopted to highlight spatial regions that are critical for accurate grasp detection. These informative regions mainly include object boundaries, potential grasp centers, contact surfaces, and slender structural areas that support reasonable gripper placement. The spatial attention mapping is formulated as follows:

(19) $$M_{s}\left(X_{\mathrm{sh}}\right)=\sigma \left[{\mathrm{Conv}}_{2}\left(\delta \left(\mathrm{BN}\left({\mathrm{Conv}}_{1}\left(X_{\mathrm{sh}}\right)\right)\right)\right)\right].$$

The final refined feature is obtained by adaptive spatial weighting:

(20) $$Y=X_{\mathrm{sh}}⊙M_{s}\left(X_{\mathrm{sh}}\right)$$

where ${\mathrm{Conv}}_{1}\left(\cdot \right)$ and ${\mathrm{Conv}}_{2}\left(\cdot \right)$ represent two convolutional layers, BN(·) denotes batch normalization, δ(·) denotes the nonlinear activation function, and σ(·) denotes the sigmoid function for generating spatial attention weights.

The spatial attention branch does not change the formal name of the operation; rather, its functional role is to refine the spatial feature representation by assigning higher weights to grasp-relevant regions and lower weights to background or weakly informative responses.

#### 2.4.4. Distinction from Existing Attention Mechanisms

The proposed GCSA shares the general objective of feature recalibration with existing attention mechanisms, but differs in its internal organization.

Compared with SE and ECA, which mainly model channel importance, GCSA additionally includes spatial attention to localize grasp-relevant regions. Compared with CBAM, which sequentially applies channel and spatial attention, GCSA inserts a channel shuffle operation between the two branches to explicitly promote cross-group channel interaction before spatial weighting. Compared with Shuffle Attention, GCSA adopts a sequential task-oriented process in which channel attention is used first for semantic recalibration, followed by channel shuffling and spatial attention.

Accordingly, the effectiveness of GCSA should be evaluated not only by comparing the complete module with existing attention mechanisms but also by separately examining the respective contributions of channel attention, channel shuffle, and spatial attention. These comparisons are reported in the component-level ablation and attention module experiments in Section 4.

### 2.5. Multi-Scale Feature Fusion

The hierarchical backbone outputs contain complementary information. Shallow features preserve object contours and spatial details, whereas deep features contain stronger category-level semantics. To comprehensively integrate detailed spatial cues and high-level semantic knowledge, each stage feature is unified and aggregated through a multi-scale fusion pipeline. Specifically, each hierarchical feature is first projected into a consistent channel dimension through a 1×1 convolution:

(21) $$F_{i}^{\prime }={\mathrm{Conv}}_{1\times 1}\left(F_{i}\right),\;i=1,2,3,4.$$

All mapped features are then upsampled to the spatial resolution consistent with the first-stage output to achieve spatial alignment:

(22) $${\hat{F}}_{i}=\mathrm{Up}\left(F_{i}^{\prime }\right),\;i=1,2,3,4$$

where Up(·) represents the pixel-level interpolation upsampling operation adopted in the implementation. Finally, the channel-consistent and spatially aligned multi-scale features are concatenated to form the unified fused feature representation:

(23) $$F_{\mathrm{fuse}}=\mathrm{Concat}\left({\hat{F}}_{1},{\hat{F}}_{2},{\hat{F}}_{3},{\hat{F}}_{4}\right).$$

In this work, $F_{\mathrm{fuse}}$ is uniformly defined to represent the final multi-scale fused feature and is not repeatedly assigned to other intermediate features, ensuring consistent notation usage throughout the entire network description.

### 2.6. Spatial and Channel Reconstruction Grasping Module

Multi-scale feature fusion combines fine spatial details from shallow stages with high-level semantic information from deeper stages, providing the grasping head with complementary geometric and semantic cues. However, directly aggregating features from different levels may also introduce repeated spatial responses and correlated channel representations. Such redundancy can weaken grasp-related feature discrimination and interfere with the dense prediction of grasp quality, orientation, and gripper opening. To address this issue, spatial and channel reconstruction is introduced into the grasping head in order to refine the fused representation before grasp-map prediction.

Inspired by the spatial and channel reconstruction strategy of ScConv, we design a Spatial and Channel Reconstruction Grasping Module (SCRGM). The SCRGM block in the overall network architecture of Figure 2 is detailed in Figure 4. SCRGM consists of a Spatial Reconstruction Unit (SRU), a Channel Reconstruction Unit (CRU), progressive upsampling layers, and four task-specific grasp prediction branches. The SRU separates feature responses according to their information content and performs cross reconstruction to reduce spatial redundancy, whereas the CRU reorganizes correlated channel responses through channel splitting, compression, grouped transformation, adaptive reweighting, and additive fusion. By integrating these reconstruction operations with multi-scale fused features and dense grasp prediction, SCRGM aims to preserve complementary information while producing more discriminative representations for estimating grasp quality, orientation, and gripper opening.

Given the multi-scale fused feature $F_{\mathrm{fuse}}\in R^{B\times C\times H_{f}\times W_{f}}$, the overall processing pipeline of SCRGM consists of spatial reconstruction, channel reconstruction, feature upsampling, and dense grasp prediction, which is formulated as follows:

(24) $$F_{\mathrm{sru}}=\mathrm{SRU}\left(F_{\mathrm{fuse}}\right)$$

(25) $$F_{\mathrm{cru}}=\mathrm{CRU}\left(F_{\mathrm{sru}}\right)$$

(26) $$F_{\mathrm{up}}=\mathrm{Upsample}\left(F_{\mathrm{cru}}\right)$$

(27) $$\left\{Q,C,S,W_{g}\right\}=\mathrm{Pred}\left(F_{\mathrm{up}}\right)$$

where *Q* denotes the grasp quality confidence map, *C* and *S* represent two complementary orientation-encoding feature maps, and $W_{g}$ corresponds to the predicted gripper opening width map.

#### 2.6.1. Spatial Reconstruction Unit

The multi-scale fused representation may contain repeated responses at object boundaries, background regions, and candidate grasp locations. To distinguish feature responses according to their relative information content, the SRU first performs grouped normalization and subsequently reconstructs gated feature components to suppress redundant spatial responses. Given the input feature $X=F_{\mathrm{fuse}}$, the grouped normalization operation is formulated as

(28) $$X_{\mathrm{gn}}=\mathrm{GBN}\left(X\right),$$

where GBN(·) denotes the grouped batch normalization operation implemented by GroupBatchNorm2d. The input feature is evenly divided into $G_{n}$ independent groups, and the mean and standard deviation are calculated within each group for individual feature normalization:

(29) $$\mu_{g}=\frac{1}{M}\sum_{j=1}^{M}x_{g,j}$$

(30) $$\sigma_{g}=\sqrt{\frac{1}{M-1}\sum_{j=1}^{M}{\left(x_{g,j}-\mu_{g}\right)}^{2}}$$

(31) $${\hat{x}}_{g,j}=\frac{x_{g,j}-\mu_{g}}{\sigma_{g}+\varepsilon }$$

where *M* denotes the number of elements within each group and ε represents a tiny constant to avoid numerical division-by-zero instability. The normalized features are then scaled and shifted via learnable affine parameters:

(32) $$X_{\mathrm{gn}}=\gamma ⊙\hat{X}+\beta .$$

In order to adaptively evaluate the relative importance of different feature channels, the learnable scaling parameters are normalized along the channel dimension:

(33) $$\omega =\frac{\gamma }{\sum_{i=1}^{C}\gamma_{i}}.$$

The spatial reconstruction gating weights are generated by combining normalized features and channel importance coefficients:

(34) $$R=\sigma (X_{\mathrm{gn}}⊙\omega )$$

where σ(·) denotes the sigmoid activation function for producing soft gating maps. With a fixed gating threshold τ=0.5 adopted in our implementation, the feature responses are adaptively partitioned into informative and less informative components via binary masking:

(35) $$M_{\mathrm{info}}=I\left(R\geq \tau \right),$$

(36) $$M_{\mathrm{non}}=I\left(R<\tau \right),$$

(37) $$X_{1}=M_{\mathrm{info}}⊙X_{\mathrm{gn}},$$

(38) $$X_{2}=M_{\mathrm{non}}⊙X_{\mathrm{gn}}.$$

Instead of directly discarding low-information features or simply concatenating the two branched features, the SRU splits both $X_{1}$ and $X_{2}$ into two equal channel subsets for complementary cross-reconstruction:

(39) $$X_{11},X_{12}=\mathrm{Split}\left(X_{1}\right),$$

(40) $$X_{21},X_{22}=\mathrm{Split}\left(X_{2}\right).$$

Finally, cross-feature reconstruction and concatenation are performed to yield the spatially refined output:

(41) $$F_{\mathrm{sru}}=\mathrm{Concat}\left(X_{11}+X_{22},X_{12}+X_{21}\right).$$

This cross-reconstruction mechanism enables interaction and complementarity between high-information and low-information feature components, rather than completely discarding less dominant responses; accordingly, the SRU effectively suppresses repeated and redundant spatial activations while retaining potentially useful marginal features, thereby refining spatial representations for accurate grasp localization.

#### 2.6.2. Channel Reconstruction Unit

Although SRU reduces spatial redundancy, correlations may remain among the output channels, particularly because the fused feature originates from multiple backbone stages. To reorganize these correlated channel responses and further purify feature representations, the CRU adopts a elaborate split–squeeze–transform–fuse strategy for channel-wise feature recalibration. Given the spatially refined feature $F_{\mathrm{sru}}\in R^{B\times C\times H_{f}\times W_{f}}$, the input feature is first divided into an upper branch and a lower branch according to a predefined channel-splitting ratio α:

(42) $$X_{\mathrm{up}},X_{\mathrm{low}}=\mathrm{Split}\left(F_{\mathrm{sru}},\alpha \right)$$

where the splitting ratio is set to $\alpha =\frac{1}{2}$ in our implementation. Accordingly, the channel numbers of the two branches are formulated as

(43) $$C_{\mathrm{up}}=\alpha C,\;C_{\mathrm{low}}=C-C_{\mathrm{up}}.$$

Both branched features are compressed via 1×1 point-wise convolutions to squeeze redundant channel dimensions:

(44) $${\tilde{X}}_{\mathrm{up}}={\mathrm{PWC}}_{1}\left(X_{\mathrm{up}}\right)$$

(45) $${\tilde{X}}_{\mathrm{low}}={\mathrm{PWC}}_{2}\left(X_{\mathrm{low}}\right)$$

where the channel compression ratio $r_{s}$ is fixed to 2 for all experiments. For the upper branch, grouped convolution and point-wise convolution are deployed in parallel to capture both local structural priors and smooth channel transformations:

(46) $$Y_{1}=\mathrm{GWC}\left({\tilde{X}}_{\mathrm{up}}\right)+{\mathrm{PWC}}_{3}\left({\tilde{X}}_{\mathrm{up}}\right)$$

where the grouped convolution adopts a 3×3 kernel with two groups by default. For the lower branch, the transformed feature is concatenated with the original compressed feature to preserve multi-level channel semantics:

(47) $$Y_{2}=\mathrm{Concat}\left[{\mathrm{PWC}}_{4}\left({\tilde{X}}_{\mathrm{low}}\right),{\tilde{X}}_{\mathrm{low}}\right].$$

Subsequently, the outputs of the two branches are concatenated for comprehensive channel aggregation:

(48) $$Y=\mathrm{Concat}(Y_{1},Y_{2}).$$

To adaptively reweight interdependent channel responses and highlight task-relevant channels, global average pooling and channel-wise softmax normalization are performed to generate channel attention coefficients:

(49) $$A=\mathrm{Softmax}\left[\mathrm{GAP}(Y)\right].$$

The aggregated feature is recalibrated via element-wise channel weighting:

(50) $$\tilde{Y}=A⊙Y.$$

The weighted feature is evenly split into two channel subsets for complementary fusion:

(51) $${\tilde{Y}}_{1},{\tilde{Y}}_{2}=\mathrm{Split}\left(\tilde{Y}\right).$$

Finally, element-wise addition is adopted to obtain the channel-refined output feature:

(52) $$F_{\mathrm{cru}}={\tilde{Y}}_{1}+{\tilde{Y}}_{2}.$$

Unlike conventional channel attention modules that merely perform one-shot weighting, the proposed CRU reorganizes correlated channel features through splitting, dual-branch transformation, adaptive recalibration, and complementary fusion. This elaborate pipeline effectively eliminates channel redundancy while precisely restoring the original channel dimension *C*, yielding both spatially purified and channel-purified features for high-precision grasp prediction.

#### 2.6.3. Progressive Feature Upsampling

After spatial and channel reconstruction, the output feature remains at the reduced resolution of the fused backbone representation. However, grasp detection is formulated as dense pixel-wise prediction, requiring the model to recover a higher-resolution feature map before producing grasp quality, orientation, and gripper width outputs. To address this resolution mismatch, two transposed-convolution layers are adopted for progressive upsampling to gradually restore spatial resolution:

(53) $$F_{\mathrm{up}}^{\left(1\right)}={\mathrm{TConv}}_{1}\left(F_{\mathrm{cru}}\right),$$

(54) $$F_{\mathrm{up}}={\mathrm{TConv}}_{2}\left(F_{\mathrm{up}}^{\left(1\right)}\right).$$

In the complete grasp head implementation, batch normalization and nonlinear activation are sequentially appended after each transposed convolution. These auxiliary operations are inherently incorporated into Equations (53) and (54) in the actual network forward pass. Notably, the original ScConv architecture does not include upsampling layers; thus, the detailed hyperparameters of the two transposed convolutions, including kernel size, stride, padding, output channel dimension, normalization, and activation functions, are strictly defined by the integrated SCRGM grasping head.

#### 2.6.4. Task-Specific Grasp Prediction

After spatial and channel reconstruction, the refined feature map is progressively upsampled to recover the spatial resolution required for dense grasp prediction. The resulting feature representation is then processed by four independent task-specific prediction branches to estimate grasp quality, grasp orientation, and gripper opening. The grasp quality map is predicted as

(55) $$Q=P_{Q}\left(F_{\mathrm{up}}\right),$$

where $Q\in R^{B\times 1\times H\times W}$ evaluates the suitability of each spatial location as a grasp center. The grasp orientation is encoded via two continuous feature maps:

(56) $$C=P_{C}\left(F_{\mathrm{up}}\right)$$

(57) $$S=P_{S}\left(F_{\mathrm{up}}\right)$$

where *C* and *S* represent the cosine and sine components of the grasp angle, respectively. This continuous trigonometric representation eliminates angular discontinuity near the periodic boundary and achieves stable orientation regression. The detailed angle decoding formulation is provided in Section 3.8. The gripper opening width map is predicted as

(58) $$W_{g}=P_{W}\left(F_{\mathrm{up}}\right),$$

where $W_{g}\in R^{B\times 1\times H\times W}$ predicts the feasible opening width of the parallel gripper at each candidate pixel location. The four prediction branches share the same reconstructed and upsampled feature but adopt individual output convolution layers. This design enables the network to leverage shared grasp-aware feature patterns while satisfying the distinct regression objectives of grasp quality, orientation, and opening width. The overall grasp prediction process is unified as

(59) $$\left\{Q,C,S,W_{g}\right\}=\mathrm{Pred}\left(F_{\mathrm{up}}\right).$$

During inference, peak responses in the grasp quality map are selected as candidate grasp centers. The corresponding *C*, *S*, and $W_{g}$ values at these locations are then adopted to recover the precise grasp orientation and gripper opening width. The complete postprocessing and decoding pipeline is elaborated in Section 3.8.

#### 2.6.5. Design Rationale and Relation to ScConv

The purpose of the SCRGM is to adapt spatial and channel reconstruction to dense grasp prediction rather than using ScConv as a generic convolutional replacement. Multi-scale fusion provides complementary semantic and spatial information, but can also introduce repeated spatial responses and correlated channel features. To address these two types of redundancy, SCRGM places SRU and CRU immediately after multi-scale feature fusion and before grasp map decoding.

The resulting processing sequence is as follows: multi-scale feature fusion → SRU → CRU → progressive upsampling → task-specific grasp prediction.

SRU first performs gated feature separation and cross reconstruction to reorganize spatial responses. CRU then processes the reconstructed feature through channel splitting, compression, grouped transformation, adaptive weighting, and additive fusion. The refined representation is subsequently upsampled and mapped into four dense outputs corresponding to grasp quality, orientation, and gripper opening.

This arrangement is specifically designed for the grasping head. As opposed to directly inserting ScConv into the backbone as a general feature-processing block, SCRGM operates on fused multi-scale features and is jointly connected to the dense grasp decoder; therefore, it links feature reconstruction with the requirements of pixel-wise grasp estimation, including precise grasp center localization, continuous orientation prediction, and gripper width regression.

The individual contributions of SRU and CRU are investigated through component-level ablation experiments. Under the same backbone and training protocol, the baseline grasping head, SRU-only variant, CRU-only variant, and complete SCRGM are compared to assess the effects of spatial reconstruction, channel reconstruction, and their combination on grasp detection accuracy.

### 2.7. Multi-Task Prediction and Objective Function

Robotic manipulation in workshop environments requires not only the localization of feasible grasp configurations but also the identification of object categories for subsequent sorting and task execution. Performing these two tasks using independent networks would require duplicated feature extraction, even though object recognition and grasp detection share common visual information such as object appearance, shape, and structural characteristics. To exploit these shared representations while retaining task-specific prediction capabilities, MTGCSAFormer adopts a multi-task architecture with a shared GCSAFormer backbone and separate classification and grasp prediction branches.

Nevertheless, the two tasks impose different requirements on the learned representation: object classification primarily relies on high-level semantic features that are invariant to local spatial variations, whereas grasp detection requires spatially precise representations for estimating grasp centers, orientations, and gripper openings. Therefore, the classification and grasping tasks share the hierarchical feature extractor but employ independent prediction heads. This design allows common visual representations to be reused while reducing direct interference between task-specific output mappings.

Given an RGB input image *I*, the shared backbone extracts hierarchical features

(60) $$\left\{F_{1},F_{2},F_{3},F_{4}\right\}=B\left(I\right),$$

where $F_{4}$ contains the highest-level semantic representation and is used by the classification branch, while the multi-scale features $\left\{F_{1},F_{2},F_{3},F_{4}\right\}$ are fused and processed by SCRGM for dense grasp prediction.

The overall multi-task output is expressed as

(61) $$\left\{O_{\mathrm{cls}},Q,C,S,W_{g}\right\}=M\left(I\right),$$

where $O_{\mathrm{cls}}$ denotes the classification output, *Q* is the grasp quality map, *C* and *S* represent the grasp orientation components, and $W_{g}$ denotes the gripper opening map.

#### 2.7.1. Object Classification Branch

Object-category prediction primarily depends on high-level semantic information. Therefore, the deepest backbone feature $F_{4}$ is used as the input to the classification branch rather than the spatially reconstructed feature used for grasp prediction.

The classification output is obtained as

(62) $$O_{\mathrm{cls}}=H_{\mathrm{cls}}\left(F_{4}\right),$$

where $H_{\mathrm{cls}}\left(\cdot \right)$ denotes the classification head.

Specifically, the deepest feature is first normalized and then aggregated using global average pooling:

(63) $$z=\mathrm{GAP}\left[\mathrm{Norm}(F_{4})\right]$$

where $z\in R^{C_{4}}$ denotes the global semantic representation.

The category logits are subsequently obtained using a linear classifier:

(64) $$O_{\mathrm{cls}}=W_{\mathrm{cls}}z+b_{\mathrm{cls}}$$

where $W_{\mathrm{cls}}$ and $b_{\mathrm{cls}}$ denote the learnable parameters of the classification layer.

The predicted probability of category *k* is calculated using the softmax function:

(65) $$p_{k}=\frac{\exp(O_{\mathrm{cls},k})}{\sum_{j=1}^{K}\exp\left(O_{\mathrm{cls},j}\right)}$$

where *K* denotes the number of object categories.

For a ground-truth one-hot category label *y*, the classification objective is defined using cross-entropy loss:

(66) $$L_{\mathrm{cls}}=-\frac{1}{B}\sum_{i=1}^{B}\sum_{k=1}^{K}y_{i,k}\log p_{i,k}$$

where *B* denotes the batch size.

#### 2.7.2. Grasp Prediction Objective

Unlike object classification, grasp detection is formulated as a dense prediction task. The grasping branch predicts four spatial maps corresponding to grasp quality, two orientation components, and gripper opening:

(67) $$\left\{Q,C,S,W_{g}\right\}=H_{\mathrm{grasp}}\left(F_{\mathrm{fuse}}\right)$$

where $H_{\mathrm{grasp}}\left(\cdot \right)$ represents the multi-scale fusion, spatial/channel reconstruction, progressive upsampling, and task-specific prediction operations described in Section 3.5 and Section 3.6.

For the grasp-quality map, the regression objective is defined as

(68) $$L_{Q}=\frac{1}{N}\sum_{i=1}^{N}{\left(Q_{i}-Q_{i}^{*}\right)}^{2},$$

where $Q_{i}$ and $Q_{i}^{*}$ denote the predicted and ground-truth grasp quality values, respectively, and *N* denotes the number of evaluated spatial elements.

The grasp orientation is represented by two continuous components. Their regression loss is defined as

(69) $$L_{\mathrm{ori}}=\frac{1}{N}\sum_{i=1}^{N}\left[{\left(C_{i}-C_{i}^{*}\right)}^{2}+{\left(S_{i}-S_{i}^{*}\right)}^{2}\right],$$

where $C_{i}^{*}$ and $S_{i}^{*}$ denote the ground-truth orientation representations.

The gripper opening regression loss is

(70) $$L_{W}=\frac{1}{N}\sum_{i=1}^{N}{\left(W_{g,i}-W_{g,i}^{*}\right)}^{2}.$$

Thus, the complete grasp detection objective is written as

(71) $$L_{\mathrm{grasp}}=\lambda_{Q}L_{Q}+\lambda_{\mathrm{ori}}L_{\mathrm{ori}}+\lambda_{W}L_{W},$$

where $\lambda_{Q}$, $\lambda_{\mathrm{ori}}$, and $\lambda_{W}$ control the relative contributions of the three grasp-related objectives.

#### 2.7.3. Multi-Task Objective

Because classification and grasp detection emphasize different properties of the shared representation, directly assigning equal importance to the two tasks may lead to competition during optimization. Therefore, a weighted multi-task objective is adopted to control the contribution of the auxiliary classification task:

(72) $$L_{\mathrm{total}}=L_{\mathrm{grasp}}+\lambda_{\mathrm{cls}}L_{\mathrm{cls}},$$

or equivalently,

(73) $$L_{\mathrm{total}}=\lambda_{Q}L_{Q}+\lambda_{\mathrm{ori}}L_{\mathrm{ori}}+\lambda_{W}L_{W}+\lambda_{\mathrm{cls}}L_{\mathrm{cls}},$$

where $\lambda_{\mathrm{cls}}$ determines the contribution of object classification to the joint optimization process.

Small $\lambda_{\mathrm{cls}}$ places greater emphasis on spatially precise grasp prediction, whereas a larger value increases the influence of semantic classification on the shared backbone. Therefore, $\lambda_{\mathrm{cls}}$ represents an explicit trade-off between the two learning objectives rather than being treated as a fixed architectural constant.

The classification branch is introduced to provide object-category information together with grasp configurations through a shared perception model. It is not assumed that the auxiliary classification objective necessarily improves grasp detection accuracy, since the two tasks may produce partially competing gradients in the shared backbone. Consequently, the influence of classification supervision is examined through task-specific ablation experiments and classification loss weight sensitivity analysis.

#### 2.7.4. Analysis of Task Interaction

The multi-task formulation is motivated by the functional requirement for simultaneous object recognition and grasp estimation rather than by an assumption of uniformly positive transfer between the two tasks. Although both tasks benefit from common visual cues, their optimization objectives differ. Classification encourages semantic discrimination and invariance to local spatial changes, whereas grasp detection requires sensitivity to local geometry and spatial position.

Accordingly, the effect of task interaction is evaluated by comparing a grasp-only configuration with the complete multi-task configuration under the same backbone, data partition, and training protocol. In addition, different values of $\lambda_{\mathrm{cls}}$ are evaluated to investigate the sensitivity of grasp detection to classification supervision. These experiments are used to determine whether the auxiliary task produces positive, neutral, or negative transfer and to identify an appropriate balance between category recognition and grasp prediction.

This analysis provides a more explicit interpretation of the multi-task architecture: the classification branch supplies semantic category prediction required for object-aware robotic manipulation, while the grasping branch provides spatially precise grasp configurations. Their shared backbone enables common visual representations to be reused, whereas separate prediction heads and loss weighting preserve task-specific learning requirements.

### 2.8. Grasp Decoding and Eye-in-Hand Coordinate Transformation

MTGCSAFormer predicts grasp configurations in the image coordinate system, whereas robotic manipulation requires a grasp pose expressed in the robot base coordinate system. To bridge this gap, the dense grasp maps are first decoded into an explicit planar grasp representation. The depth value corresponding to the predicted grasp center is then combined with the calibrated camera intrinsic parameters to recover the three-dimensional grasp point in the camera coordinate system. Finally, the eye-in-hand calibration result and the current end-effector pose are used to transform the grasp point into the robot base coordinate system.

MTGCSAFormer is an RGB-based perception network. RGB images are used for object classification and planar grasp prediction, while depth information is not involved in network feature extraction or grasp map prediction. Instead, the aligned depth image is used after network inference to recover the metric three-dimensional position of the predicted grasp point. Therefore, RGB information is used for grasp perception, whereas depth-assisted geometric localization is employed during physical robot execution.

The complete perception-to-execution process consists of RGB image acquisition, two-dimensional grasp prediction, depth association, three-dimensional position recovery, eye-in-hand coordinate transformation, and robot grasp execution.

#### 2.8.1. Planar Grasp Decoding

For a parallel-jaw gripper, a planar grasp configuration is represented by the grasp center coordinates, grasp orientation, gripper opening, and grasp quality score:

(74) $$g_{2D}=\left(u,v,\theta ,w,q\right)$$

where (u,v) denotes the grasp center coordinates in the image plane, θ represents the in-plane grasp orientation, *w* denotes the gripper opening, and *q* represents the grasp quality score.

Given the predicted grasp quality map *Q*, the grasp center is selected from the position with the maximum response:

(75) $$\left(u^{*},v^{*}\right)=\underset{u,v}{\arg\max}\;Q\left(u,v\right).$$

The corresponding grasp quality score is

(76) $$q^{*}=Q\left(u^{*},v^{*}\right).$$

The orientation components at the selected grasp center are obtained from the predicted orientation maps:

(77) $$C^{*}=C\left(u^{*},v^{*}\right),$$

(78) $$S^{*}=S\left(u^{*},v^{*}\right).$$

For a parallel-jaw gripper, the orientations θ and θ+π describe the same physical grasp. Therefore, the orientation is represented using double-angle encoding:

(79) C=cos(2θ),S=sin(2θ).

The predicted grasp orientation is recovered as

(80) $$\theta^{*}=\frac{1}{2}atan2\left(S^{*},C^{*}\right).$$

The corresponding gripper opening is obtained from the predicted width map:

(81) $$w^{*}=W_{g}\left(u^{*},v^{*}\right).$$

The decoded planar grasp is consequently expressed as

(82) $$g_{2D}^{*}=\left(u^{*},v^{*},\theta^{*},w^{*},q^{*}\right).$$

This decoding procedure converts the dense grasp maps into an explicit image-plane grasp representation for subsequent three-dimensional localization.

#### 2.8.2. Depth-Assisted Three-Dimensional Grasp Point Recovery

The predicted grasp center $\left(u^{*},v^{*}\right)$ represents a two-dimensional pixel position and cannot independently determine the physical location of the grasp point. Therefore, the depth value corresponding to the predicted grasp center is obtained from the depth image aligned with the RGB image:

(83) $$Z_{C}=D\left(u^{*},v^{*}\right)$$

where D(·) denotes the aligned depth map and $Z_{C}$ represents the distance of the grasp point along the camera optical axis.

The calibrated camera intrinsic matrix is defined as follows:

(84) $$K=\left[\begin{array}{ccc} f_{x} & 0 & c_{x}\\ 0 & f_{y} & c_{y}\\ 0 & 0 & 1 \end{array}\right]$$

where $f_{x}$ and $f_{y}$ denote the focal lengths in the horizontal and vertical directions, respectively, and $\left(c_{x},c_{y}\right)$ denotes the principal point.

According to the pinhole camera model, the projection relationship between a three-dimensional point in the camera coordinate system and its corresponding pixel coordinate is

(85) $$u=f_{x}\frac{X_{C}}{Z_{C}}+c_{x},\;v=f_{y}\frac{Y_{C}}{Z_{C}}+c_{y}.$$

Given the predicted pixel coordinate and its corresponding depth, the three-dimensional grasp-point coordinates are recovered as

(86) $$X_{C}=\frac{\left(u^{*}-c_{x}\right)Z_{C}}{f_{x}},$$

(87) $$Y_{C}=\frac{\left(v^{*}-c_{y}\right)Z_{C}}{f_{y}},$$

(88) $$Z_{C}=D\left(u^{*},v^{*}\right).$$

The back-projection process can also be expressed in matrix form:

(89) $$\left[\begin{array}{c} X_{C}\\ Y_{C}\\ Z_{C} \end{array}\right]=Z_{C}K^{-1}\left[\begin{array}{c} u^{*}\\ v^{*}\\ 1 \end{array}\right].$$

The homogeneous coordinate of the recovered grasp point is

(90) $${\tilde{P}}_{C}=\left[\begin{array}{c} X_{C}\\ Y_{C}\\ Z_{C}\\ 1 \end{array}\right].$$

In this process, the camera intrinsic parameters determine the direction of the projection ray corresponding to the predicted pixel, whereas the depth value determines the metric position along this ray. Their combination enables the predicted two-dimensional grasp center to be recovered as a three-dimensional point in the camera coordinate system.

#### 2.8.3. Eye-in-Hand Calibration

An eye-in-hand configuration is adopted to establish the geometric relationship between the visual system and the robotic manipulator [44,45]. The camera is rigidly mounted on the robot end effector such that their relative pose remains unchanged during robot motion. A calibration board is fixed in the workspace, while the robot is moved to multiple poses so that the camera observes the calibration board from different viewpoints.

Let {B}, {E}, {C}, and {T} denote the robot base, end-effector, camera, and calibration board coordinate systems, respectively. The coordinate transformation chain for a point expressed in the calibration board coordinate system is

(91) PB=TEBTCETTCPT,

where TEB denotes the transformation from the end-effector coordinate system to the robot base coordinate system, TCE denotes the transformation from the camera coordinate system to the end-effector coordinate system, and TTC denotes the transformation from the calibration board coordinate system to the camera coordinate system.

For each calibration image, the transformation between the calibration board coordinate system and the camera coordinate system is estimated using the detected calibration board feature points, the calibrated camera intrinsic parameters, and the PnP method. Their projection relationship is as follows:

(92) $$s\left[\begin{array}{c} u\\ v\\ 1 \end{array}\right]=K\left[\begin{array}{cc} R_{CT} & t_{CT} \end{array}\right]\left[\begin{array}{c} X_{T}\\ Y_{T}\\ Z_{T}\\ 1 \end{array}\right]$$

where *s* denotes the projective scale factor while $R_{CT}$ and $t_{CT}$ respectively denote the rotation matrix and translation vector from the calibration board coordinate system to the camera coordinate system.

The corresponding homogeneous transformation is

(93) TTC=RCTtCT0T1.

For two robot poses *i* and *j*, the relative motion of the end effector is represented as

(94) Aij=TE,iB−1TE,jB.

The corresponding relative camera motion obtained from the calibration board observations is

(95) Bij=TT,iCTT,jC−1.

The eye-in-hand calibration problem is formulated as

(96) $$A_{ij}X=XB_{ij}.$$

The unknown transformation is represented as

(97) X=TCE=RECtEC0T1.

Multiple robot poses and calibration board observations are used to solve Equation (96), obtaining the fixed geometric relationship between the camera and the robot end effector.

#### 2.8.4. Transformation to the Robot Base Coordinate System

After eye-in-hand calibration, the transformation TCE remains fixed because the camera is rigidly attached to the end-effector. At the instant of image acquisition, the robot controller provides the current transformation from the end-effector coordinate system to the robot base coordinate system:

(98) TEB=RBEtBE0T1.

The recovered grasp point is first transformed from the camera coordinate system to the end-effector coordinate system:

(99) P˜E=TCEP˜C.

It is subsequently transformed from the end-effector coordinate system to the robot base coordinate system:

(100) P˜B=TEBP˜E.

Combining the two transformations gives

(101) P˜B=TEBTCEP˜C.

Therefore, the instantaneous camera-to-base transformation is

(102) TCB=TEBTCE.

The resulting grasp point in the robot base coordinate system is as follows:

(103) $${\tilde{P}}_{B}=\left[\begin{array}{c} X_{B}\\ Y_{B}\\ Z_{B}\\ 1 \end{array}\right]$$

where $\left(X_{B},Y_{B},Z_{B}\right)$ specifies the target grasp position for the robotic manipulator.

Because the camera moves together with the end effector, TCE remains constant after hand–eye calibration, whereas TEB varies with the current robot pose. Therefore, the end-effector pose recorded at the instant of image acquisition is used to maintain consistency between the visual observation and the coordinate transformation.

#### 2.8.5. Grasp Pose Generation and Robot Execution

In addition to the three-dimensional grasp position, the predicted grasp orientation and gripper opening must be converted into executable robot commands. The grasp angle $\theta^{*}$ describes the in-plane rotation of the parallel-jaw gripper.

The rotation component of the camera-to-base transformation is

(104) $$R_{BC}=R_{BE}R_{EC}.$$

Let $d_{C}$ denote the grasp-direction vector expressed in the camera coordinate system. Its corresponding direction in the robot base coordinate system is obtained as

(105) $$d_{B}=R_{BC}d_{C}=R_{BE}R_{EC}d_{C}.$$

The transformed grasp direction is used to determine the rotation of the end-effector around the grasp approach axis. The remaining orientation components are specified according to the predefined approach direction and the geometric relationship among the camera, end-effector, and parallel-jaw gripper.

The predicted grasp width is converted into the physical gripper opening command according to

(106) $$w_{\mathrm{cmd}}=G\left(w^{*}\right),$$

where G(·) denotes the calibrated mapping from the predicted width representation to the physical gripper opening.

The complete executable grasp pose is expressed as

(107) $$G_{B}=\left(X_{B},Y_{B},Z_{B},R_{B},w_{\mathrm{cmd}}\right),$$

where $R_{B}$ denotes the desired end-effector orientation in the robot base coordinate system.

During execution, the manipulator first moves to a pre-grasp pose above the target position, then approaches the predicted grasp point along the predefined approach direction. After reaching the target grasp pose, the gripper closes and the object is lifted. For object sorting tasks, the category predicted by the classification branch is further used to determine the corresponding placement region.

## 3. Experiments and Results

### 3.1. Experimental Datasets

Planar grasp detection datasets provide annotated grasp configurations for evaluating the ability of a model to localize feasible grasp positions and orientations. Among the publicly available benchmarks, the Cornell Grasping Dataset [46] and the Jacquard Dataset [47] are two commonly used datasets for planar grasp detection. Their main characteristics are summarized in Table 1.

In this study, the Cornell Grasping Dataset is used to evaluate the grasp detection performance of the proposed MTGCSAFormer and to facilitate comparison with existing methods. In addition, the General Tools and Parts in Workshop Dataset (GTPWD) is constructed to evaluate joint object category recognition and grasp detection in workshop-oriented robotic manipulation scenarios. Unlike the Cornell dataset, which is used primarily for grasp detection, GTPWD provides both object category labels and grasp annotations, enabling evaluation of the proposed multi-task framework.

#### 3.1.1. Cornell Grasping Dataset

The Cornell Grasping Dataset is a widely used benchmark for planar robotic grasp detection [46]. It contains 885 RGB-D images of 240 different objects acquired from multiple viewpoints. Each image has a spatial resolution of 640×480 pixels and is associated with multiple grasp annotations.

The dataset contains 5110 positive grasp rectangles and 2909 negative grasp rectangles. A positive rectangle represents a feasible parallel-jaw grasp, whereas a negative rectangle denotes an unsuitable grasp configuration. Consistent with common planar grasp detection settings, only positive grasp annotations are used as supervision in the present study.

Each grasp rectangle is defined by its center position, orientation, width, and height. During evaluation, the original grasp rectangles provided by the dataset are retained. In particular, the annotated rectangle dimensions are used directly rather than replacing the grasp width with a fixed average value. This treatment ensures that the reported results are compatible with the standard rectangle-based evaluation protocol.

Because the Cornell dataset contains a relatively limited number of images and multiple views of the same object, the data partitioning strategy has a significant influence on the reported performance. To provide a transparent evaluation, both image-wise and object-wise 80:20 partitioning protocols are adopted. Under the image-wise protocol, individual images are assigned to different folds, and different views of the same object may appear in both the training and test sets. Under the object-wise protocol, all images belonging to the same physical object are assigned to the same fold, evaluating the generalization to previously unseen objects.

The dataset partitioning is completed before data augmentation; therefore, augmented versions of a training image cannot appear in the validation or test subsets. To reduce overfitting, the training images are augmented using random rotation, cropping, translation, and scaling. The corresponding grasp annotations are transformed using the same geometric operations.

**Figure 5:**
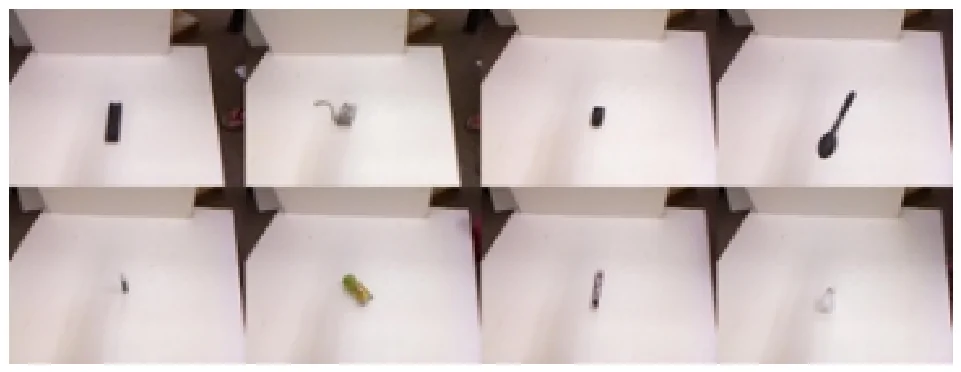
Representative RGB images from the Cornell Grasping Dataset.

**Figure 6:**
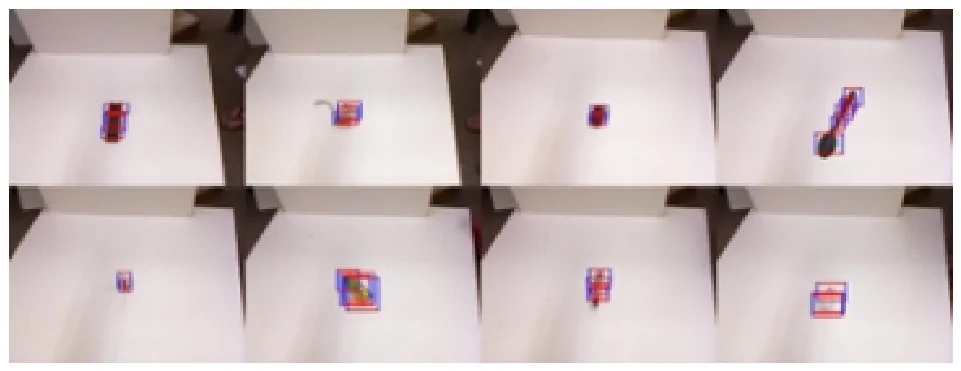
Examples of valid grasp rectangle annotations in the Cornell Grasping Dataset. Multiple feasible grasp configurations may be associated with the same object image.

Representative RGB images from the Cornell Grasping Dataset are shown in Figure 5, and examples of the corresponding grasp rectangle annotations are presented in Figure 6.

#### 3.1.2. General Tools and Parts in Workshop Dataset

The General Tools and Parts in Workshop Dataset (GTPWD) is constructed to evaluate robotic grasp detection and object recognition in workshop-oriented scenarios. The dataset contains eight categories of commonly used tools and mechanical parts: wrenches, screwdrivers, pliers, utility knives, hex keys, bolts, nuts, and drill bits.

Each category contains ten distinct physical objects. Images of each object are acquired under three different backgrounds and multiple object poses. Through repeated image acquisition under different arrangements, the dataset contains a total of 2400 RGB images. Each image is associated with an object category label and one or more planar grasp annotations.

The images were acquired using an Intel RealSense D455 RGB-D camera at a resolution of 640×480 pixels. Aligned RGB and depth streams were recorded during acquisition; however, only RGB images were used for network training and inference. The aligned depth stream was used only in the physical robotic system after network inference to recover the three-dimensional position of the predicted grasp center.

**Figure 7:**
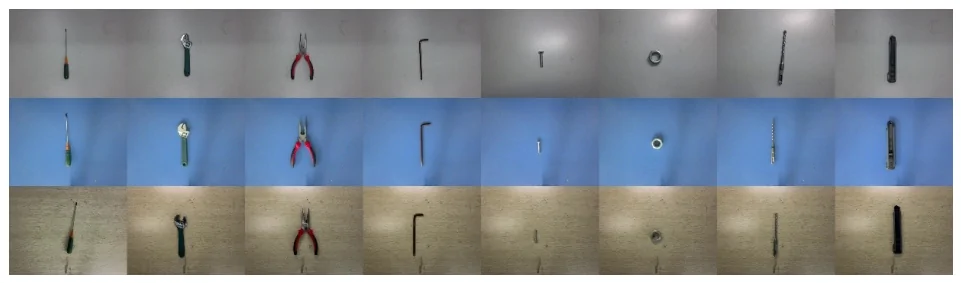
Representative RGB samples from the General Tools and Parts in Workshop Dataset (GTPWD), including workshop tools and mechanical parts captured with an Intel RealSense D455 camera under different backgrounds and object poses.

The dataset is designed to capture appearance and geometric variations commonly encountered in workshop manipulation, including changes in object pose, background texture, illumination, and object scale. Representative samples from GTPWD are shown in Figure 7.

For each image, the object category is assigned according to the corresponding tool or part type. The planar grasp annotation specifies the grasp center, in-plane orientation, and required gripper opening for a parallel-jaw gripper. An annotation is considered valid when the selected contact region lies within the object boundary, the required opening is within the physical range of the gripper, and the selected grasp is mechanically feasible.

To improve annotation reliability, the annotations are checked in two stages. The initial annotations are first generated according to the predefined grasp labeling criteria. A second manual verification is then performed to identify geometrically invalid or mechanically unstable grasps. Annotations are revised or removed when the grasp center lies outside the feasible contact region, the grasp orientation causes unstable contact, or the required gripper opening exceeds the allowable range.

The complete dataset is first divided into training and test portions at a ratio of 80:20. The training portion is then divided into training and validation subsets at a ratio of 9:1. Consequently, 72%, 8%, and 20% of the complete dataset are used for training, validation, and testing, respectively. Images acquired from the same object sequence are assigned to the same subset in order to reduce the possibility of highly similar observations appearing in both the training and test data. The resulting dataset partition is summarized in Table 2.

To assess annotation consistency, a subset of 200 images is independently rechecked. The consistency is evaluated using grasp center deviation, orientation deviation, grasp rectangle IoU, and the percentage of annotations satisfying the predefined agreement criterion. The corresponding results are reported in Table 3.

The relatively small deviations in grasp center and orientation together with the high rectangle overlap and agreement rate indicate that the annotation procedure provides consistent grasp labels for model training and evaluation.

### 3.2. Implementation Details

All experiments are implemented using PyTorch2.1.0 on a workstation equipped with an AMD R9-7945HX processor and an NVIDIA RTX 4060 GPU with 8 GB of memory.

The reported inference time is measured for single-image network execution at an input resolution of 300×300 pixels on the same RTX 4060 GPU. The average inference time is approximately 7 ms per image. This measurement covers the neural network forward pass and excludes RGB-D image acquisition, depth lookup, three-dimensional coordinate recovery, robot communication, motion planning, and physical gripper execution.

The input RGB images are resized to 300×300 pixels. The grasp center coordinates and gripper width annotations are scaled according to the same resizing ratios, while the grasp orientation remains unchanged under isotropic resizing.

The GCSAFormer-S24 configuration is used as the backbone. Its four stages contain 4, 4, 12, and 4 blocks, respectively, and their corresponding channel dimensions are 64, 128, 320, and 512. Compatible backbone parameters are initialized from the PoolFormer-S24 checkpoint [28], whereas the GCSA modules and grasp prediction head are initialized separately.

The 1000 iterations per epoch correspond to the number of augmented mini-batches processed during training, rather than the number of original training images. Given a batch size of 4, each epoch contains 4000 dynamically augmented training samples, which are randomly generated through stochastic data augmentation during iterative sampling. Validation and test data are excluded from this augmentation process in order to ensure unbiased evaluation. The detailed training hyper-parameters are listed in Table 4.

### 3.3. Evaluation Metrics

#### 3.3.1. Representation of Manipulator Grasp Poses

**Figure 8:**
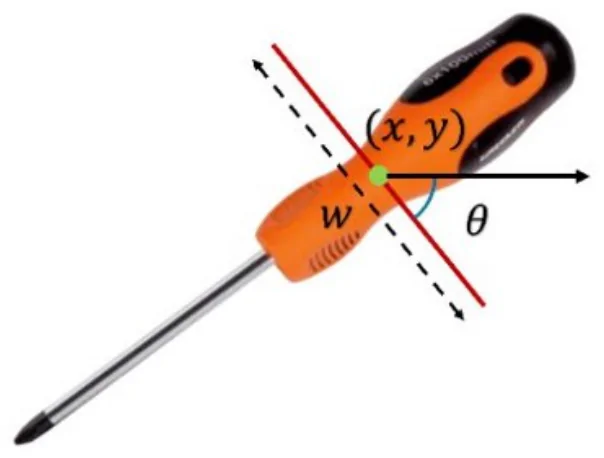
Grasp pose representation of manipulators.

When a manipulator performs grasping tasks, defining an appropriate representation for object grasp position and orientation is essential. It directly determines whether the manipulator can successfully grasp objects and exerts a profound influence on grasping efficiency and accuracy. An ideal representation should facilitate calculations for deep learning algorithms and enable the robotic end gripper to precisely adjust its posture according to the predicted grasp information. A suitable grasp representation can effectively improve the generalization capability of robotic grasping networks. Such a representation is compatible with various network architectures and manipulators, supports reliable object grasping under complex and dynamic environments, and enhances the adaptability and robustness of robots operating in diverse scenarios. The widely adopted grasp representation is the five-dimensional grasp rectangle proposed by Lenz et al. [46]:

(108) $$\begin{array}{c} G=\left\{x,y,h,w,\theta \right\} \end{array}$$

where (x,y) is the center coordinate of the grasp rectangle and *h*, *w*, and θ respectively denote the height, width, and rotation angle of the grasp rectangle. The height of the grasp rectangle is determined by the gripper opening range and has the smallest impact on grasp representation; therefore, the grasp representation can be simplified as the following formula, as illustrated in Figure 8:

(109) $$\begin{array}{c} G=\left\{x,y,w,\theta \right\}. \end{array}$$

Furthermore, the grasp quality *q*, which characterizes the probability of successful grasping at each grasp candidate, is introduced into the grasp representation. Its value ranges from 0 to 1. The final grasp representation is expressed as

(110) $$\begin{array}{c} G=\left\{x,y,q,w,\theta \right\}. \end{array}$$

#### 3.3.2. Grasp Detection Evaluation Metric

The proposed network represents a grasp using the five-dimensional formulation

(111) $$G_{p}=\left\{x_{p},y_{p},q_{p},w_{p},\theta_{p}\right\},$$

where $\left(x_{p},y_{p}\right)$ denotes the predicted grasp center, $q_{p}$ is the grasp quality score, $w_{p}$ represents the predicted gripper opening, and $\theta_{p}$ denotes the predicted in-plane grasp orientation. Unlike methods that directly regress the four vertices or all geometric dimensions of a grasp rectangle, the proposed representation does not explicitly predict the short-side length *h* of the rectangle.

However, the commonly adopted Cornell evaluation protocol determines grasp correctness according to the angular difference and the intersection over union between a predicted grasp rectangle and the annotated grasp rectangles. Therefore, the predicted grasp pose must first be converted into an oriented rectangle before the overlap criterion can be calculated.

The predicted evaluation rectangle is defined as

(112) $$R_{p}=R\left(x_{p},y_{p},w_{p},h_{e},\theta_{p}\right),$$

where R(·) denotes the operation that converts the predicted grasp pose into an oriented rectangle. The rectangle center and orientation are determined by $\left(x_{p},y_{p}\right)$ and $\theta_{p}$, respectively. The long-side length is determined by the predicted gripper opening $w_{p}$, while the short-side length $h_{e}$ is fixed as

(113) $$h_{e}=30\;\mathrm{pixels}.$$

The value of 30 pixels is obtained from the average short-side length of the positive grasp rectangles in the Cornell Grasping Dataset. Because the short-side dimension mainly describes the local contact region along the gripper-finger direction and has a smaller influence on the executable grasp configuration than the grasp center, orientation, and opening width, it is not included as an independent network output. The fixed value is used only to reconstruct a rectangle for evaluation, and does not participate in network training or robot grasp execution.

Let

(114) $$c_{p}=\left[\begin{array}{c} x_{p}\\ y_{p} \end{array}\right]$$

denote the center of the predicted grasp rectangle. The unit vectors along the long-side and short-side directions are defined as

(115) $$e_{w}=\left[\begin{array}{c} \cos\theta_{p}\\ \sin\theta_{p} \end{array}\right],\;e_{h}=\left[\begin{array}{c} -\sin\theta_{p}\\ \cos\theta_{p} \end{array}\right].$$

The four vertices of the reconstructed grasp rectangle are calculated as

(116) $$p_{1}=c_{p}+\frac{w_{p}}{2}e_{w}+\frac{h_{e}}{2}e_{h},$$

(117) $$p_{2}=c_{p}+\frac{w_{p}}{2}e_{w}-\frac{h_{e}}{2}e_{h},$$

(118) $$p_{3}=c_{p}-\frac{w_{p}}{2}e_{w}-\frac{h_{e}}{2}e_{h},$$

(119) $$p_{4}=c_{p}-\frac{w_{p}}{2}e_{w}+\frac{h_{e}}{2}e_{h}.$$

Following the standard Cornell rectangle-based protocol [17,46], a predicted grasp is regarded as correct when the reconstructed rectangle satisfies both the orientation difference criterion and the rectangle overlap criterion with respect to at least one ground-truth grasp rectangle.

For a parallel-jaw gripper, the orientations θ and θ+π describe the same physical grasp. Therefore, the angular difference is calculated using the periodic formulation

(120) $$\Delta \theta =\min\left(\left|\theta_{p}-\theta_{g}\right|,\pi -\left|\theta_{p}-\theta_{g}\right|\right),$$

where $\theta_{g}$ denotes the orientation of a ground-truth grasp rectangle. The orientation criterion is defined as

(121) $$\Delta \theta <30^{\circ }.$$

The rectangle overlap is evaluated using the standard intersection-over-union metric:

(122) $$\mathrm{IoU}=\frac{\left|R_{p}\cap R_{g}\right|}{\left|R_{p}\cup R_{g}\right|}$$

where $R_{p}$ denotes the reconstructed predicted rectangle and $R_{g}$ denotes a ground-truth grasp rectangle. The overlap criterion is defined as

(123) IoU>0.25.

For an image containing multiple valid ground-truth grasp rectangles, the predicted grasp is considered correct if there exists at least one annotation satisfying both Equations (121) and (123). The overall grasp-detection accuracy is calculated as

(124) $${\mathrm{Acc}}_{\mathrm{grasp}}=\frac{N_{\mathrm{correct}}}{N_{\mathrm{test}}}\times 100\%,$$

where $N_{\mathrm{correct}}$ denotes the number of correctly predicted test images and $N_{\mathrm{test}}$ denotes the total number of evaluated images.

### 3.4. Experimental Results on the Cornell Grasping Dataset

The Cornell Grasping Dataset was used to evaluate the grasp detection accuracy and generalization ability of MTGCSAFormer. The complete dataset was divided into training and test sets at a ratio of 80:20. The training portion was further divided into a training subset and a validation subset at a ratio of 9:1. All data augmentation operations were applied only to the training subset after data partitioning.

Two commonly used partitioning protocols were adopted: image-wise splitting (IW) and object-wise splitting (OW). Under the IW protocol, all images were randomly assigned to the training and test sets; therefore, different images of the same physical object could appear in both sets. This protocol mainly evaluates the adaptability of the model to changes in object position, orientation, and viewpoint.

Under the OW protocol, all images belonging to the same physical object were assigned to the same subset. Consequently, the objects contained in the test set did not appear during training. In this way, the OW protocol provides a more stringent evaluation of the model’s generalization ability to previously unseen objects.

The two partitioning protocols are summarized in Table 5.

For both protocols, 90% of the training portion was used for model optimization and the remaining 10% for validation. The test set remained independent throughout model training, hyperparameter selection, and data augmentation.

#### 3.4.1. Comparison with Existing Grasp Detection Methods

MTGCSAFormer was compared with representative planar grasp detection methods published in recent years. The compared approaches include dense pixel-wise grasp predictors, encoder–decoder networks, feature fusion methods, and lightweight grasp detection models. The results are presented in Table 6.

As shown in Table 6, MTGCSAFormer achieves a grasp-detection accuracy of 99.5% under both the IW and OW protocols. Under the IW protocol, the proposed method outperforms AFFGA-Net by 0.41 percentage points and exceeds the other compared methods by a larger margin.

More importantly, MTGCSAFormer maintains an accuracy of 99.5% under the stricter OW protocol. Compared with the IW setting, the OW setting prevents the same physical object from appearing in both the training and test sets. Therefore, high OW accuracy indicates that the model can transfer the learned grasp-related representations to previously unseen object instances.

MTGCSAFormer requires an average inference time of approximately 7 ms per 300×300 input image on the reported RTX 4060 platform. This value denotes the neural network forward time rather than a speed metric or the duration of the complete robotic grasping cycle. Its inference time is comparable to that of MSG-ConvNet and lower than those of GG-CNN, SE-ResUNet, AFFGA-Net, CLRG, SISG-Net, UFGNet, and LPGNet. Although Efficient-Grasp reports a slightly shorter inference time of 6 ms, its IW and OW accuracy results are 1.7 percentage points lower than those of MTGCSAFormer. These results indicate that the proposed model provides a favorable balance between grasp detection accuracy and measured inference time under the reported hardware configuration.

It should be noted that MTGCSAFormer uses only RGB images as the input to the grasp prediction network. The depth image does not participate in network feature extraction or dense grasp map prediction. Depth information is used after network inference to recover the three-dimensional position of the predicted grasp center for physical robotic execution.

#### 3.4.2. Evaluation Rectangle Construction

MTGCSAFormer predicts a grasp pose using the representation

(125) $$G_{p}=\left\{x_{p},y_{p},q_{p},w_{p},\theta_{p}\right\},$$

where $\left(x_{p},y_{p}\right)$ denotes the grasp center, $q_{p}$ represents the grasp-quality score, $w_{p}$ denotes the gripper opening, and $\theta_{p}$ represents the grasp orientation. The model does not directly regress the four vertices or the short-side dimension of a Cornell-style grasp rectangle.

To apply the rectangle-based evaluation protocol, the predicted grasp pose is converted into an oriented rectangle:

(126) $$R_{p}=R\left(x_{p},y_{p},w_{p},h_{e},\theta_{p}\right),$$

where the rectangle center, long-side length, and orientation are determined by $\left(x_{p},y_{p}\right)$, $w_{p}$, and $\theta_{p}$, respectively. The short-side length is set to

(127) $$h_{e}=30\;\mathrm{pixels}.$$

The value of 30 pixels corresponds to the average short-side length of the positive grasp rectangles in the Cornell Grasping Dataset. It is used only to reconstruct an evaluation rectangle, and is not included as a network prediction target or robot control parameter.

The orientation difference between the predicted grasp and a ground-truth grasp is calculated as

(128) $$\Delta \theta =\min\left(\left|\theta_{p}-\theta_{g}\right|,\pi -\left|\theta_{p}-\theta_{g}\right|\right),$$

where $\theta_{g}$ denotes the orientation of the ground-truth rectangle. The periodic formulation accounts for the fact that the orientations θ and θ+π correspond to the same parallel-jaw grasp.

The predicted grasp satisfies the orientation criterion when

(129) $$\Delta \theta <30^{\circ }.$$

The overlap between the reconstructed predicted rectangle $R_{p}$ and the ground-truth rectangle $R_{g}$ is calculated using the standard intersection-over-union definition:

(130) $$\mathrm{IoU}=\frac{\left|R_{p}\cap R_{g}\right|}{\left|R_{p}\cup R_{g}\right|}.$$

The overlap criterion is defined as

(131) IoU>0.25.

For an image containing multiple valid grasp annotations, the prediction is considered correct when at least one ground-truth rectangle simultaneously satisfies Equations (129) and (131).

#### 3.4.3. Sensitivity to the Evaluation-Rectangle Short Side

Because the short-side length $h_{e}$ is introduced only for rectangle-based evaluation, its influence on the reported grasp accuracy was further examined. The network predictions were kept unchanged, while $h_{e}$ was varied from 20 to 40 pixels. The corresponding results are presented in Table 7.

The results show that the IW accuracy varies from 99.1% to 99.5%, while the OW accuracy varies from 99.0% to 99.5%, for maximum variations of 0.4 and 0.5 percentage points, respectively. This limited variation indicates that the evaluation results are not excessively sensitive to a narrowly selected rectangle dimension.

The best result is obtained using $h_{e}=30$ pixels, which is consistent with the average short-side length of the Cornell positive grasp annotations. This result supports the use of 30 pixels as the default value for converting the predicted grasp pose into an oriented evaluation rectangle.

#### 3.4.4. Qualitative Results

**Figure 9:**
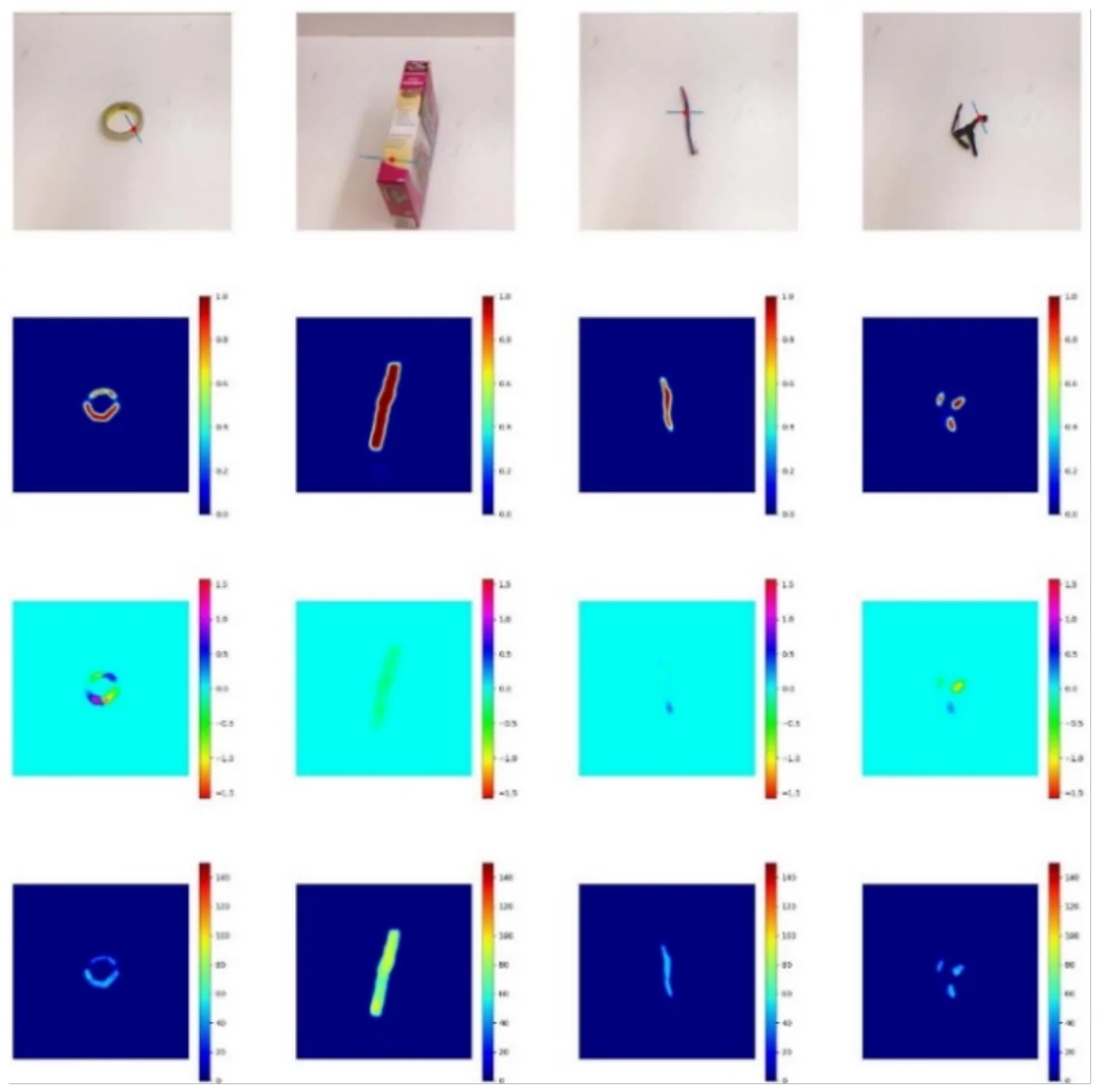
Representative grasp detection results on the Cornell Grasping Dataset. The upper row shows the predicted grasp poses, while the lower rows show the grasp quality, orientation, and gripper width maps.

Representative grasp detection results on the Cornell Grasping Dataset are shown in Figure 9. For each example, the upper row presents the predicted grasp pose, while the lower rows show the grasp quality, orientation, and gripper width maps.

In the grasp quality maps, regions with higher response values represent locations with a higher probability of successful grasping. The orientation maps encode the required rotation of the parallel-jaw gripper at each candidate position, while the width maps represent the corresponding gripper opening.

The qualitative results demonstrate that MTGCSAFormer can identify feasible grasp regions for objects with different shapes, scales, and orientations. For elongated objects, the predicted grasp directions generally align with the local geometric structure. For compact objects, the network tends to select stable contact regions near the central part of the object. These observations are consistent with the quantitative results in Table 6.

### 3.5. Multi-Task Evaluation on GTPWD

The General Tools and Parts in Workshop Dataset (GTPWD) was used to evaluate the ability of MTGCSAFormer to simultaneously perform planar grasp detection and object classification in workshop-oriented robotic manipulation scenarios. Unlike the Cornell Grasping Dataset, the GTPWD provides both grasp annotations and object-category labels, enabling the grasping and classification tasks to be evaluated within a unified framework.

All results in this subsection were obtained on the held-out GTPWD test set. The test images were not used for model optimization, hyperparameter selection, early stopping, or data augmentation.

The complete dataset was divided into training and test sets at a ratio of 80:20. The training portion was further divided into training and validation subsets at a ratio of 9:1. All data augmentation operations were applied exclusively to the training subset. The same dataset partition, backbone configuration, and training settings were used for all experiments presented in this section.

#### 3.5.1. Joint Grasp Detection and Object Classification

To investigate the influence of the object classification task on grasp detection, two configurations were compared. The first configuration contained only the grasp-prediction branch and was optimized using the grasp detection objective. The second configuration was the complete multi-task MTGCSAFormer, in which the grasping and classification branches shared the GCSAFormer backbone and were jointly optimized.

The experimental results are presented in Table 8.

As shown in Table 8, the grasp-only configuration achieves a grasp detection accuracy of 98.5%. When the classification branch is introduced, the complete multi-task model achieves a grasp accuracy of 98.0% and a classification accuracy of 99.5%.

The grasp accuracy of the multi-task model is 0.5 percentage points lower than that of the grasp-only model. This result indicates that the auxiliary classification objective does not improve grasp detection accuracy and instead introduces a mild negative transfer effect.

The negative transfer can be explained by the different representation requirements of the two tasks. Object classification primarily relies on category-discriminative semantic information and favors representations that remain relatively invariant to changes in object position and local geometry. In contrast, grasp detection requires accurate preservation of spatial information, including object boundaries, grasp centers, orientations, contact regions, and gripper openings. Therefore, joint optimization may produce partially competing gradients in the shared backbone.

Nevertheless, the multi-task architecture remains meaningful for the target robotic manipulation scenario. The grasping branch determines how the object should be grasped, whereas the classification branch determines the semantic category required for subsequent sorting and placement. Therefore, the classification task is introduced to provide an additional function required by the robotic system rather than to serve solely as an auxiliary objective for improving grasp accuracy.

**Figure 10:**
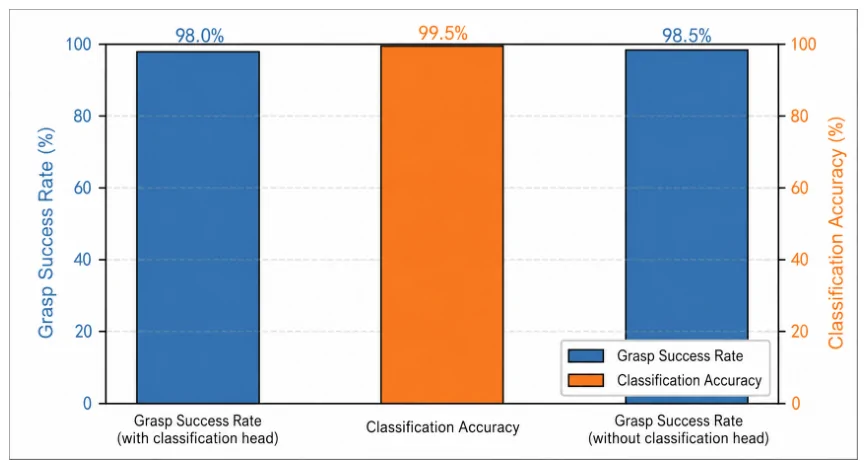
Comparison of grasp detection and classification performance under the grasp-only and multi-task configurations on GTPWD.

The comparison between the grasp-only and multi-task configurations is further illustrated in Figure 10.

#### 3.5.2. Classification Loss Weight Sensitivity

To further analyze the interaction between grasp detection and object classification, the influence of the classification loss weight was investigated. The total multi-task objective is defined as

(132) $$L_{\mathrm{total}}=L_{\mathrm{grasp}}+\lambda_{\mathrm{cls}}L_{\mathrm{cls}},$$

where $L_{\mathrm{grasp}}$ denotes the grasp detection loss, $L_{\mathrm{cls}}$ denotes the classification loss, and $\lambda_{\mathrm{cls}}$ controls the contribution of classification supervision during joint optimization.

When $\lambda_{\mathrm{cls}}=0$, the network is optimized only for grasp detection. As $\lambda_{\mathrm{cls}}$ increases, the influence of category-level semantic supervision on the shared representation becomes stronger. The corresponding experimental results are presented in Table 9.

When $\lambda_{\mathrm{cls}}$ increases from 0 to 0.10, the classification accuracy increases to 99.5%, whereas the grasp detection accuracy gradually decreases from 98.5% to 98.0%. This trend confirms that strengthening classification supervision changes the shared representation in favor of category-level semantic discrimination.

When $\lambda_{\mathrm{cls}}$ is increased beyond 0.10, the classification accuracy remains approximately unchanged, while the grasp accuracy continues to decrease. In particular, increasing $\lambda_{\mathrm{cls}}$ from 0.10 to 0.50 reduces grasp accuracy from 98.0% to 97.2% without producing a meaningful improvement in classification performance.

These results indicate that an excessively large classification loss weight causes the classification objective to dominate the shared feature learning process, thereby weakening the spatially precise information required for grasp prediction. In contrast, an excessively small value does not provide sufficient semantic supervision for reliable category prediction.

Accordingly, $\lambda_{\mathrm{cls}}=0.10$ is selected for the complete MTGCSAFormer. This setting achieves a grasp detection accuracy of 98.0% and a classification accuracy of 99.5%, providing a practical balance between geometric grasp estimation and semantic object recognition.

It should be emphasized that the selected loss weight does not maximize grasp accuracy. The highest grasp accuracy is obtained when the classification task is removed. The value of $\lambda_{\mathrm{cls}}=0.10$ is selected because the target workshop application requires both grasp and category outputs.

#### 3.5.3. Qualitative Multi-Task Results

**Figure 11:**
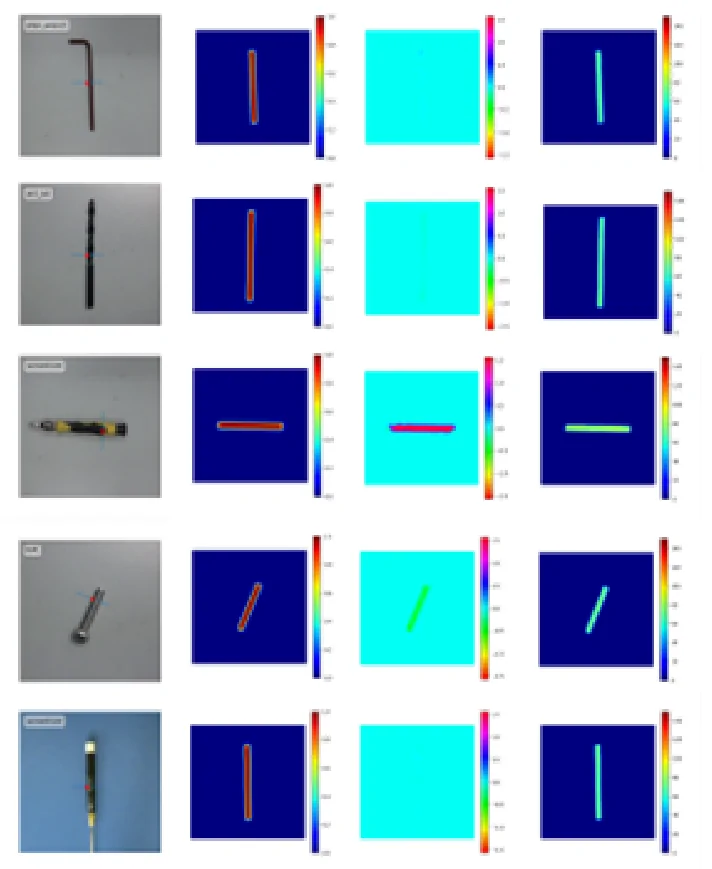
Representative multi-task prediction results of MTGCSAFormer on GTPWD. The network simultaneously predicts the object category and the corresponding planar grasp configuration.

Representative multi-task prediction results on GTPWD are shown in Figure 11. Each example presents the predicted object category and the corresponding planar grasp configuration.

The qualitative results demonstrate that MTGCSAFormer can jointly recognize workshop objects and estimate feasible grasp configurations under different poses and backgrounds. For elongated objects such as screwdrivers, wrenches, hex keys, and drill bits, the predicted grasp orientation generally follows the local geometric direction of the object. For relatively compact parts such as nuts and bolts, the predicted grasp centers are concentrated around stable contact regions.

The grasping and classification branches therefore provide complementary outputs for robotic manipulation. The grasping branch supplies geometric information for robot motion planning and gripper control, whereas the classification branch provides semantic information for determining the subsequent sorting or placement destination.

### 3.6. Ablation Studies

To investigate the contributions of the GCSAFormer backbone and the Spatial and Channel Reconstruction Grasping Module (SCRGM), a series of ablation experiments were conducted on the GTPWD. All experimental configurations used the same dataset partition, input resolution, data augmentation strategy, optimization parameters, and multi-task loss setting. Only the backbone or grasping head under investigation was changed.

The ablation experiments were organized into four parts. First, the contributions of GCSAFormer and SCRGM were evaluated using four backbone and grasping head combinations. Second, the internal channel attention, channel shuffle, and spatial attention operations of GCSA were investigated separately. Third, GCSA was compared with representative attention mechanisms. Finally, the contributions of SRU, CRU, and multi-scale feature fusion were evaluated.

#### 3.6.1. Overall Ablation of GCSAFormer and SCRGM

Four configurations were constructed by combining the PoolFormer or GCSAFormer backbone with the original grasping head (GG) or SCRGM. The PoolFormer + GG configuration was regarded as the baseline, whereas GCSAFormer + SCRGM represented the complete model.

The four configurations are defined as follows:

- **PoolFormer + GG**: The baseline model using the PoolFormer backbone and the original grasping head.
- **PoolFormer + SCRGM**: The PoolFormer backbone combined with the spatial and channel reconstruction grasping module.
- **GCSAFormer + GG**: The GCSAFormer backbone combined with the original grasping head.
- **GCSAFormer + SCRGM**: The complete model containing both the GCSAFormer backbone and SCRGM.

**Figure 12:**
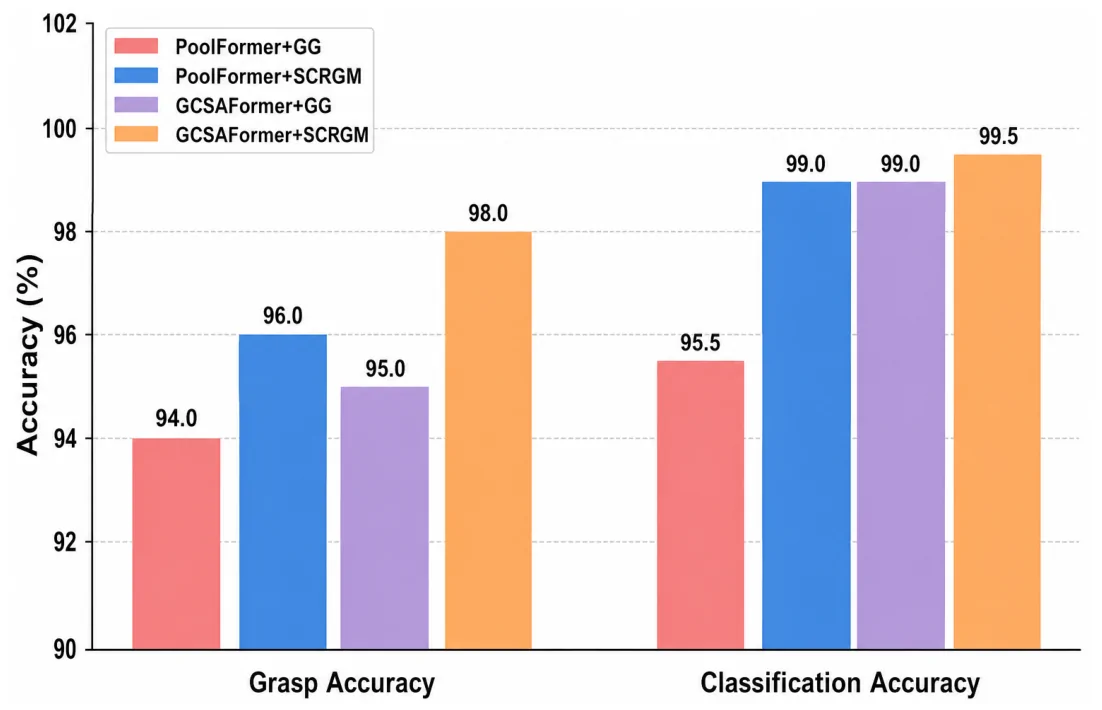
Ablation results of different backbone and grasping head configurations on GTPWD.

The quantitative results are presented in Table 10 and Figure 12.

As shown in Table 10, the PoolFormer + GG baseline achieves a grasp detection accuracy of 94.0% and a classification accuracy of 95.5%.

Replacing the original grasping head with SCRGM while retaining the PoolFormer backbone increases grasp accuracy from 94.0% to 96.0%, an improvement of 2.0 percentage points. The classification accuracy also increases from 95.5% to 99.0%. This improvement indicates that spatial and channel reconstruction refines the feature representation provided to the dense grasp-prediction branches.

Although SCRGM is primarily designed for grasp prediction, its introduction also affects the gradients propagated through the shared backbone during multi-task optimization. Thus, the observed classification improvement should be interpreted as an empirical effect of joint training rather than as evidence that SCRGM directly performs object classification.

Replacing PoolFormer with GCSAFormer while retaining the original GG head increases the grasp accuracy from 94.0% to 95.0%. More notably, the classification accuracy increases from 95.5% to 99.0%. This result indicates that the channel and spatial feature-refinement operations in GCSAFormer substantially improve category-discriminative semantic representations while also providing a moderate benefit for grasp prediction.

The complete GCSAFormer + SCRGM configuration achieves the highest grasp accuracy of 98.0% and classification accuracy of 99.5%. Compared with the PoolFormer + GG baseline, the complete model improves grasp accuracy by 4.0 percentage points and classification accuracy by 4.0 percentage points.

Compared with GCSAFormer + GG, introducing SCRGM increases grasp accuracy from 95.0% to 98.0%, corresponding to an improvement of 3.0 percentage points. Classification accuracy increases only slightly from 99.0% to 99.5%. This result is consistent with the task-specific role of SCRGM, which has the main purpose of refining fused features for dense grasp prediction.

Compared with PoolFormer + SCRGM, replacing PoolFormer with GCSAFormer increases grasp accuracy from 96.0% to 98.0% and classification accuracy from 99.0% to 99.5%. This result demonstrates that GCSAFormer remains beneficial even when the reconstruction-based grasping head is already employed.

Overall, GCSAFormer and SCRGM contribute at different stages of the multi-task architecture. GCSAFormer primarily improves the shared hierarchical representation, with a particularly clear benefit for object classification. SCRGM primarily improves grasp detection by reorganizing spatial and channel responses before the prediction of grasp quality, orientation, and gripper opening. Their joint use provides the best performance for both tasks.

#### 3.6.2. Component-Level Ablation of GCSA

The overall ablation results demonstrate that replacing PoolFormer with GCSAFormer improves both grasp detection and object classification. However, this comparison does not identify the contribution of each operation within GCSA. Therefore, channel attention (CA), channel shuffle (CS), and spatial attention (SA) were evaluated separately.

SCRGM and the remaining network architecture were retained in all configurations. The configuration without CA, CS, or SA corresponds to the baseline backbone combined with SCRGM. The results are presented in Table 11.

The configuration without channel attention, channel shuffle, or spatial attention achieves a grasp accuracy of 96.0% and a classification accuracy of 99.0%. These results correspond to the PoolFormer + SCRGM configuration in Table 10.

Introducing channel attention increases grasp accuracy from 96.0% to 96.7%. This improvement suggests that adaptive channel recalibration helps emphasize channels containing grasp-related geometry and semantic information.

Spatial attention alone increases grasp accuracy to 96.5%. Although the improvement is slightly smaller than that produced by channel attention, it indicates that spatial weighting helps emphasize regions associated with feasible contact positions.

Combining channel attention and spatial attention increases grasp accuracy to 97.5%. This result demonstrates that channel and spatial refinement provide complementary information. Channel attention emphasizes informative feature dimensions, whereas spatial attention identifies grasp-relevant locations within these features.

When channel shuffle is introduced together with channel attention, the grasp accuracy reaches 97.2%. The channel shuffling operation facilitates feature exchange among different channel groups, reducing the isolation introduced by group-wise feature processing.

The complete GCSA configuration, containing channel attention, channel shuffling, and spatial attention, achieves the highest grasp accuracy of 98.0% and classification accuracy of 99.5%. Compared with the channel-and-spatial attention configuration without channel shuffling, the complete structure improves grasp accuracy by 0.5 percentage points.

These results indicate that the performance of GCSA does not originate from a single attention operation; instead, channel recalibration, channel redistribution, and spatial refinement provide complementary effects when organized sequentially.

#### 3.6.3. Comparison with Existing Attention Mechanisms

To determine whether the improvement of GCSA results merely from adding an attention module, GCSA was compared with SE, ECA, CBAM, and Shuffle Attention. Each alternative attention module replaced GCSA at the same positions in the backbone, while SCRGM and all other experimental settings were kept unchanged.

The comparison results are shown in Table 12.

All evaluated attention mechanisms improve grasp performance compared with the configuration without attention. SE and ECA achieve grasp accuracy results of 96.5% and 96.7%, respectively, confirming that channel recalibration is beneficial for grasp-related feature extraction.

CBAM, which combines channel and spatial attention, achieves 97.2%. Shuffle Attention further increases the grasp accuracy to 97.5%, indicating that grouped feature interaction can provide additional benefits.

GCSA achieves the highest grasp accuracy of 98.0% and classification accuracy of 99.5%. It outperforms CBAM by 0.8 percentage points in grasp accuracy and Shuffle Attention by 0.5 percentage points.

These comparisons do not imply that channel attention, channel shuffle, or spatial attention are independently new concepts. The contribution of GCSA lies specifically in organizing these established operations into a channel recalibration–channel redistribution–spatial refinement sequence suited to the hierarchical features used for dense grasp prediction.

#### 3.6.4. Component-Level Ablation of SCRGM

To identify the contributions of spatial reconstruction and channel reconstruction, the Spatial Reconstruction Unit (SRU) and Channel Reconstruction Unit (CRU) were evaluated separately. GCSAFormer was retained in all configurations.

Four grasping head configurations were considered: the original grasping head without reconstruction, an SRU-only head, a CRU-only head, and the complete SCRGM containing both units. The results are presented in Table 13.

Without SRU or CRU, the GCSAFormer + GG configuration achieves a grasp accuracy of 95.0% and a classification accuracy of 99.0%.

Introducing SRU alone increases the grasp accuracy to 96.2%. This improvement suggests that gated separation and cross-reconstruction reduce repeated spatial responses and help to preserve informative regions for grasp localization.

CRU alone achieves a grasp accuracy of 96.7%, outperforming the SRU-only configuration by 0.5 percentage points. This result indicates that channel splitting, compression, grouped transformation, adaptive weighting, and feature reconstruction effectively reorganize correlated channel information.

When the SRU and CRU are combined in sequence, the complete SCRGM achieves 98.0% grasp accuracy and 99.5% classification accuracy. Compared with the reconstruction-free head, the SCRGM improves grasp accuracy by 3.0 percentage points.

The improvement produced by the complete module is larger than those of the individual units, indicating that spatial and channel reconstruction address different types of redundancy in the fused representation.

The reconstruction principles of SRU and CRU are related to ScConv. The task-specific contribution of SCRGM lies in integrating these units with multi-scale fused features, progressive upsampling, and dense prediction branches for grasp quality, orientation, and gripper opening.

#### 3.6.5. Ablation of Multi-Scale Feature Fusion

The GCSAFormer backbone generates four hierarchical feature maps, $F_{1}$, $F_{2}$, $F_{3}$, and $F_{4}$, from shallow to deep stages. To investigate the contribution of different feature levels, progressively increasing numbers of hierarchical features were provided to SCRGM.

GCSA and SCRGM were retained in all configurations. The results are shown in Table 14.

Using only the deepest feature, $F_{4}$, achieves a grasp accuracy of 96.6%. Although $F_{4}$ contains strong semantic information, its reduced spatial resolution limits the preservation of object boundaries and local grasp geometry.

Combining $F_{3}$ and $F_{4}$ increases grasp accuracy to 97.1%, indicating that intermediate-resolution features provide additional spatial information. Further adding $F_{2}$ increases the accuracy to 97.6%.

The complete fusion of all four feature levels achieves the highest grasp accuracy of 98.0% and classification accuracy of 99.5%. The progressive improvements demonstrate that shallow and deep features provide complementary information. Shallow features preserve boundaries and local geometry, whereas deep features provide semantic and contextual representations.

The relatively small but consistent increase at each stage indicates that multi-scale feature fusion incrementally refines the representation rather than acting as the sole source of the final performance improvement.

#### 3.6.6. Summary of the Ablation Results

The ablation experiments demonstrate that GCSAFormer and SCRGM provide different but complementary contributions. Replacing PoolFormer with GCSAFormer mainly improves the shared semantic representation, increasing classification accuracy from 95.5% to 99.0% when the original grasping head is retained. Replacing GG with SCRGM primarily improves dense grasp prediction, increasing grasp accuracy from 94.0% to 96.0% when PoolFormer is retained.

The complete GCSAFormer + SCRGM configuration achieves the highest grasp and classification accuracies of 98.0% and 99.5%, respectively. The component-level experiments further show that channel attention, channel shuffle, and spatial attention each contribute to GCSA, while SRU and CRU provide complementary spatial and channel reconstruction within SCRGM.

Comparisons with SE, ECA, CBAM, and Shuffle Attention indicate that the specific organization adopted in GCSA provides a moderate but consistent improvement over related attention mechanisms. Similarly, progressively incorporating hierarchical backbone features improves grasp performance, supporting the use of multi-scale feature fusion.

Overall, the final performance of MTGCSAFormer results from backbone-level channel–spatial feature refinement, hierarchical feature aggregation, and grasp head-level spatial/channel reconstruction rather than from a single architectural component.

### 3.7. Input Resolution Analysis

The input resolution directly affects the amount of spatial information available for grasp center localization, orientation estimation, and gripper opening prediction. A relatively low resolution may remove fine object boundaries and narrow graspable regions, whereas an excessively high resolution increases the input size without necessarily producing a corresponding improvement in grasp accuracy.

To justify the use of 300×300 images, MTGCSAFormer was evaluated using five input resolutions: 224×224, 256×256, 300×300, 320×320, and 384×384. The same dataset partition, backbone configuration, loss weights, augmentation strategy, and training schedule were used in all experiments. Only the input resolution was changed.

All RGB images were resized using bilinear interpolation. The corresponding grasp center coordinates and gripper opening annotations were scaled using the same resizing ratio. Because isotropic resizing was adopted, the grasp orientation remained unchanged.

The experimental results are presented in Table 15.

As shown in Table 15, increasing the input resolution from 224×224 to 256×256 improves the grasp accuracy from 96.9% to 97.5%. Increasing the resolution to 300×300 further raises grasp accuracy to 98.0% and classification accuracy to 99.5%.

The improvement at 300×300 can be attributed to the preservation of finer object contours, narrow graspable regions, and local orientation cues. These spatial details are particularly important for dense prediction of the grasp center and gripper opening.

When the input resolution is increased from 300×300 to 320×320, grasp accuracy improves by only 0.1 percentage points, whereas classification accuracy remains unchanged. A further increase to 384×384 does not provide an additional grasp improvement and increases classification accuracy by only 0.1 percentage points.

These results indicate that performance begins to saturate when the input resolution exceeds 300×300. Therefore, 300×300 is selected as the default input resolution because it preserves sufficient spatial detail for grasp prediction while avoiding an unnecessarily large input representation.

It should be noted that this experiment evaluates only the influence of image resolution on prediction accuracy. Since computational complexity is not analyzed separately in this study, the selected resolution is justified primarily by the observed saturation of grasp and classification performance rather than by a claim of minimum computational cost.

### 3.8. Physical Robotic Grasping Experiments

To evaluate whether the grasp configurations predicted by MTGCSAFormer can be reliably converted into executable robot actions, physical grasping experiments were conducted using an AUBO-i3 robotic manipulator. The experimental system adopts an eye-in-hand configuration consisting of an Intel RealSense D455 RGB-D camera and an EG2-4C2 parallel gripper mounted on the robot end-effector.

The physical experiments were designed to evaluate the complete perception-to-execution pipeline, including RGB-based grasp prediction, depth-assisted three-dimensional localization, eye-in-hand coordinate transformation, robot motion planning, and gripper execution.

The trials were conducted under controlled single-target conditions so that the evaluation could focus on grasp prediction, depth-assisted localization, camera–robot transformation, and robot execution. Multi-object clutter additionally requires instance-level target association, inter-object occlusion handling, collision avoidance, grasp-order planning, and iterative scene updating; these factors were not systematically evaluated in the present physical experiments.

#### 3.8.1. Experimental Platform

**Figure 13:**
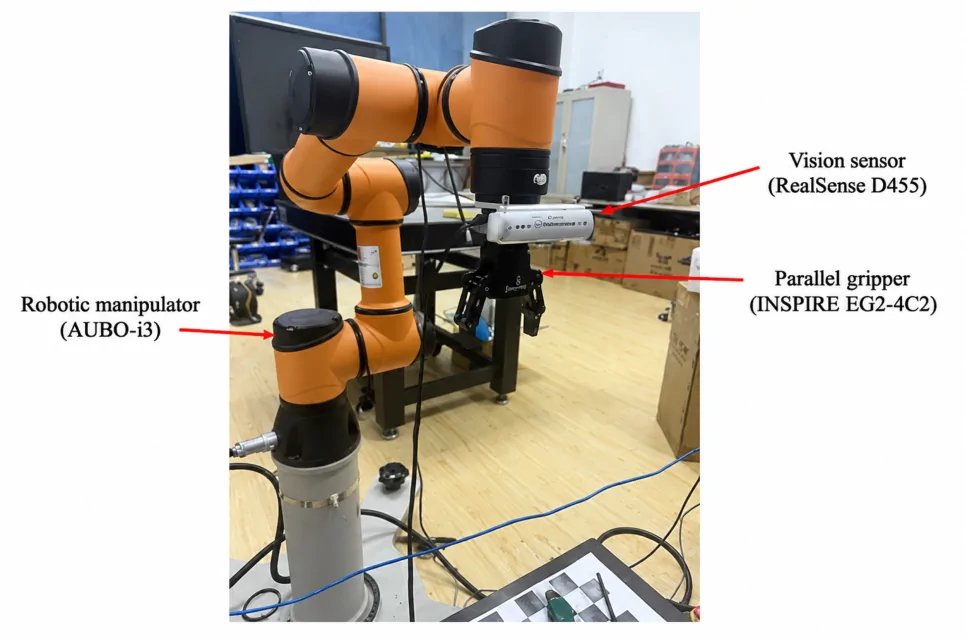
Physical robotic grasping platform consisting of an AUBO-i3 manipulator, an eye-in-hand Intel RealSense D455 RGB-D camera, and an EG2-4C2 parallel gripper.

The robotic grasping platform is shown in Figure 13. The main hardware components included the AUBO-i3 six-degrees-of-freedom manipulator, the Intel RealSense D455 RGB-D camera, and the EG2-4C2 parallel gripper.

The camera is rigidly attached to the robot end effector such that the relative transformation between the camera coordinate system and the end-effector coordinate system remains fixed after hand–eye calibration. During operation, the robot controller provides the current transformation from the end-effector coordinate system to the robot base coordinate system.

MTGCSAFormer uses the RGB image as the input for object classification and planar grasp prediction. The aligned depth image is not used for network feature extraction. Instead, the depth value corresponding to the predicted grasp center is used after inference to recover the metric three-dimensional position of the grasp point.

#### 3.8.2. Perception-to-Execution Procedure

For each grasping trial, the robot first moves to a predefined observation pose and acquires aligned RGB and depth images. The RGB image is resized to 300×300 pixels and provided to MTGCSAFormer.

The network predicts the planar grasp representation

(133) $$G_{p}=\left\{x_{p},y_{p},q_{p},w_{p},\theta_{p}\right\},$$

where $\left(x_{p},y_{p}\right)$ denotes the predicted grasp center, $q_{p}$ denotes the grasp quality score, $w_{p}$ represents the gripper opening, and $\theta_{p}$ represents the in-plane grasp orientation.

The depth value corresponding to the predicted grasp center is obtained from the aligned depth image:

(134) $$Z_{C}=D\left(x_{p},y_{p}\right),$$

where D(·) denotes the aligned depth map.

Using the calibrated camera intrinsic parameters, the predicted pixel coordinate and its depth are converted into the three-dimensional camera coordinate system:

(135) $$X_{C}=\frac{\left(x_{p}-c_{x}\right)Z_{C}}{f_{x}},\;Y_{C}=\frac{\left(y_{p}-c_{y}\right)Z_{C}}{f_{y}},$$

where $f_{x}$ and $f_{y}$ denote the camera focal lengths and $\left(c_{x},c_{y}\right)$ is the principal point.

The recovered grasp point is represented in homogeneous coordinates as

(136) $${\tilde{P}}_{C}=\left[\begin{array}{c} X_{C}\\ Y_{C}\\ Z_{C}\\ 1 \end{array}\right].$$

The eye-in-hand calibration result provides the fixed transformation TCE from the camera coordinate system to the end-effector coordinate system. The robot controller provides the current transformation TEB from the end-effector coordinate system to the robot base coordinate system. Therefore, the grasp point in the robot base coordinate system is calculated as

(137) P˜B=TEBTCEP˜C.

The predicted grasp orientation is transformed according to the rotational relationship among the camera, end-effector, and robot base coordinate systems. The predicted opening width is converted into the physical gripper command according to the calibrated gripper mapping.

After obtaining the executable grasp pose, the robot performs the following sequence: moving to a pre-grasp pose, approaching the target along the predefined direction, adjusting the gripper orientation, closing the gripper, lifting the object, and transferring it to the corresponding placement region according to the predicted object category.

#### 3.8.3. Experimental Objects and Success Criterion

The physical experiments included workshop tools and mechanical parts represented in the GTPWD as well as additional daily objects used to examine the transfer ability of the system beyond the primary dataset.

A grasp was considered successful when all of the following conditions were satisfied:

1. The gripper reached the predicted grasp region without collision.
2. The target object was securely enclosed by the gripper.
3. The object was lifted from the supporting surface.
4. The object remained in the gripper during the predefined lifting motion.

A trial was considered unsuccessful when the gripper missed the target, contacted an unstable region, failed to lift the object, or lost the object during lifting.

The physical grasp success rate was calculated as

(138) $$SR=\frac{N_{\mathrm{success}}}{N_{\mathrm{attempt}}}\times 100\%,$$

where $N_{\mathrm{success}}$ denotes the number of successful trials and $N_{\mathrm{attempt}}$ denotes the total number of grasp attempts.

#### 3.8.4. Physical Grasping Results

The physical grasping results are summarized in Table 16.

The robotic system successfully completed 76 of 80 grasp attempts, corresponding to an overall grasp success rate of 95.0%. For workshop tools and mechanical parts, 39 of 40 attempts were successful, resulting in a success rate of 97.5%. For additional daily objects, 37 of 40 attempts were successful, corresponding to a success rate of 92.5%.

The higher success rate for workshop tools and parts can be attributed to their closer similarity to the objects represented in GTPWD. The lower success rate for daily objects indicates that changes in object appearance, material, geometry, and distribution of the graspable region can affect transfer to objects outside the primary training distribution.

**Figure 14:**
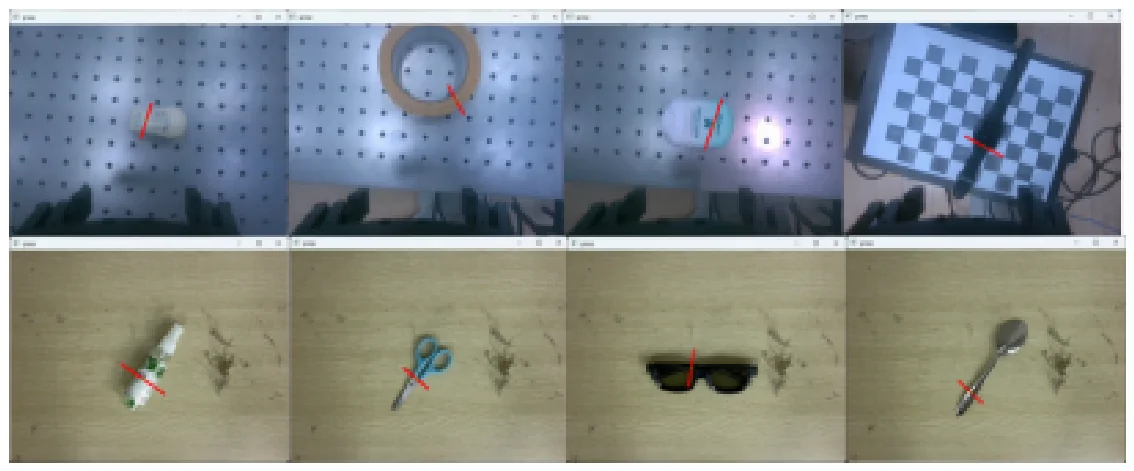
Representative physical grasping results obtained under controlled single-target conditions for workshop tools, mechanical parts, and daily objects.

Representative physical grasping results are shown in Figure 14.

The successful cases demonstrate that the predicted planar grasp center, orientation, and gripper opening can be converted into executable robot commands through depth-assisted localization and eye-in-hand coordinate transformation.

The input example used in the network architecture illustration is intended to explain the perception flow and does not indicate that the reported 95.0% physical success rate was obtained in densely cluttered multi-object scenes.

The unsuccessful cases were mainly associated with four factors. First, depth values near object boundaries occasionally exhibited instability, causing errors in three-dimensional position recovery. Second, reflective or low-texture surfaces produced unreliable depth measurements. Third, some predicted grasp centers were located near mechanically unstable object regions. Finally, object slippage occasionally occurred after gripper closure because of limited friction or unsuitable contact geometry.

These observations demonstrate that physical grasp success depends not only on the accuracy of the two-dimensional grasp prediction but on depth quality, camera calibration, hand–eye calibration, robot positioning accuracy, and gripper–object interaction.

It should also be emphasized that MTGCSAFormer is an RGB-based grasp perception network, whereas the complete robotic system uses depth information after network inference for metric three-dimensional localization. Therefore, the physical results should not be interpreted as evidence that the complete robot system operates without depth information.

### 3.9. Discussion

The experimental results provide several observations regarding the effectiveness, applicability, and limitations of MTGCSAFormer.

First, the Cornell experiments demonstrate the importance of clearly defining the evaluation protocol. MTGCSAFormer predicts a grasp pose in the form of grasp center, quality, gripper opening, and orientation rather than directly regressing the four vertices of a grasp rectangle. Therefore, a rectangle must be reconstructed before applying the Cornell rectangle-based criterion. In this study, the predicted gripper opening determines one side of the evaluation rectangle, while the other side is fixed to 30 pixels according to the average dimension of the positive Cornell grasp annotations.

The intersection-over-union metric is calculated using the standard ratio of intersection area to union area. In addition, both image-wise and object-wise data partitions are reported. The image-wise protocol evaluates adaptation to viewpoint and pose variations, whereas the object-wise protocol provides a stricter assessment because the objects contained in the test set are not observed during training. Reporting both protocols reduces ambiguity and improves comparability with previous grasp detection studies.

Second, the ablation results indicate that GCSAFormer and SCRGM contribute to different stages of the multi-task network. Replacing PoolFormer with GCSAFormer increases grasp accuracy from 94.0% to 95.0% and classification accuracy from 95.5% to 99.0% when the original grasping head is retained. This result indicates that the backbone-level channel and spatial feature refinement operations are particularly beneficial for category-discriminative semantic representation.

Replacing the original grasping head with SCRGM increases grasp accuracy from 94.0% to 96.0% when the PoolFormer backbone is retained. This result supports the use of spatial and channel reconstruction before predicting the dense grasp map. When GCSAFormer and SCRGM are used jointly, the model achieves 98.0% grasp accuracy and 99.5% classification accuracy, indicating that backbone-level feature refinement and grasp head-level reconstruction provide complementary effects.

However, the individual operations used in GCSA are related to established channel attention, spatial attention, and channel shuffling strategies. Similarly, the reconstruction principles of SRU and CRU are related to ScConv. Therefore, the contributions of GCSA and SCRGM should be interpreted as task-oriented architectural integration rather than entirely new general-purpose attention or reconstruction principles. The comparison with SE, ECA, CBAM, and Shuffle Attention and the component-level ablations are included in order to clarify this relationship.

Third, the multi-task experiments reveal a mild negative transfer effect. The grasp-only configuration achieves a grasp accuracy of 98.5%, whereas the complete multi-task model achieves 98.0%. This result demonstrates that the classification task does not necessarily improve grasp prediction. Classification favors category-discriminative semantic features that are relatively invariant to local spatial changes, whereas grasp detection requires accurate preservation of object boundaries, contact regions, orientation, and gripper opening.

Because of this, the two tasks can produce partially competing optimization objectives in the shared backbone. The classification loss sensitivity experiment further shows that while increasing the classification loss weight improves classification performance, it gradually reduces grasp accuracy. The selected loss weight represents a practical compromise rather than the setting that maximizes the performance of either task independently.

Despite this negative transfer, the multi-task architecture remains useful for the target workshop application. A robotic sorting system must determine both how an object should be grasped and which placement region should be selected according to its category. The classification branch is included to provide semantic information required for downstream task execution rather than as an auxiliary branch that guarantees higher grasp accuracy.

Fourth, the role of depth information must be distinguished between network perception and physical robot execution. MTGCSAFormer uses RGB images for feature extraction, object classification, and planar grasp prediction. Depth is not used as an input modality of the neural network. However, the complete robotic system is not depth-free; after network inference, the aligned depth value at the predicted grasp center is combined with the camera intrinsic parameters to recover the corresponding three-dimensional point in the camera coordinate system.

The eye-in-hand calibration result and the current end-effector pose are then used to transform the grasp point into the robot base coordinate system. Therefore, the system should be described as RGB-based grasp perception with depth-assisted three-dimensional localization. This distinction is important because a two-dimensional RGB prediction alone cannot uniquely determine a metric three-dimensional robot target.

Fifth, the physical grasp success rate is affected by both perception and geometric execution errors. The robotic experiments achieve an overall success rate of 95.0%, but unsuccessful trials are not necessarily caused by incorrect two-dimensional grasp prediction. Depth noise, invalid depth values near object boundaries, camera intrinsic calibration error, hand–eye calibration error, robot positioning error, and gripper–object interaction can all affect the final grasp result.

Reflective and low-texture objects are particularly challenging because their depth measurements may be unstable. In addition, a geometrically valid grasp predicted in the image may still fail because of insufficient surface friction or object slippage after gripper closure. Therefore, two-dimensional grasp accuracy and physical grasp success rate should be interpreted as related but distinct evaluation measures.

Several limitations remain in the current study. First, GTPWD contains only eight object categories and is collected using a limited number of backgrounds, viewpoints, and illumination conditions. Although the dataset is designed to represent common workshop objects, its scale remains limited relative to large synthetic datasets. The generalization ability of the model under different factories, cameras, lighting conditions, and object materials has not yet been comprehensively evaluated.

Second, all physical experiments were conducted using a single AUBO-i3 platform, one RGB-D camera, and one parallel gripper. The transferability of the system to other manipulators, camera mounting configurations, and gripper geometries remains to be established.

In addition, the current physical evaluation was limited to controlled single-target scenes. The classification branch produces one image-level category prediction, and does not explicitly associate multiple grasp candidates with multiple object instances. Consequently, the reported physical success rate should not be interpreted as evidence of robust operation in densely cluttered multi-object environments. Future work will incorporate instance-level recognition, collision-aware grasp selection, grasp order planning, and iterative scene updating.

Third, the current grasp representation focuses on planar parallel-jaw grasping. It does not explicitly estimate a complete six-degrees-of-freedom grasp pose or model complex object surface geometry. As such, the approach is more suitable for objects placed on approximately horizontal supporting surfaces than for arbitrary three-dimensional clutter.

Fourth, the use of a fixed 30-pixel short-side length is necessary because the network does not directly predict this rectangle dimension. Although the sensitivity experiment indicates that performance is relatively stable around this value, the reconstructed rectangle remains an evaluation approximation rather than a complete network output. Future work may consider explicitly predicting the full rectangle geometry or adopting evaluation metrics that operate directly on the predicted grasp pose.

Fifth, the observed negative transfer suggests that completely sharing the backbone may not provide the optimal balance between classification and grasp prediction. More advanced task balancing strategies, gradient conflict mitigation, adaptive loss weighting, or partially decoupled task-specific features may improve the joint performance.

Future work will focus on expanding GTPWD to include more object categories, materials, backgrounds, illumination conditions, and camera viewpoints. Cross-camera and cross-robot evaluation will also be conducted in order to examine hardware transferability. In addition, adaptive multi-task optimization, uncertainty-aware depth processing, improved calibration error compensation, instance-level multi-object grasping, and six-degrees-of-freedom grasp prediction will be investigated in order to improve robustness in more complex industrial environments.

### 3.10. Summary of Experimental Results

The experiments conducted on the Cornell Grasping Dataset, GTPWD, and physical robotic platform provide a comprehensive evaluation of MTGCSAFormer from the perspectives of grasp accuracy, object classification, architectural effectiveness, input resolution sensitivity, and physical execution.

On the Cornell Grasping Dataset, MTGCSAFormer achieves a grasp detection accuracy of 99.5% under both the image-wise and object-wise partitioning protocols. The image-wise protocol evaluates the adaptability of the model to variations in object pose, viewpoint, and image appearance, whereas the object-wise protocol evaluates its ability to generalize to objects that are not observed during training. The consistently high performance under both protocols indicates that the learned grasp representation can accommodate both intra-object appearance variation and previously unseen object instances.

The Cornell evaluation also clarifies the relationship between the network output and the rectangle-based grasp criterion. MTGCSAFormer predicts the grasp representation G={x,y,q,w,θ} rather than directly predicting all dimensions and vertices of a grasp rectangle. Therefore, a 30-pixel short-side length, derived from the average short-side dimension of the positive Cornell grasp annotations, is used to reconstruct the predicted rectangle for IoU evaluation. The corrected intersection-over-union definition and the short-side sensitivity experiment improve the transparency of the reported evaluation protocol.

On GTPWD, the complete multi-task model achieves a grasp detection accuracy of 98.0% and an object-classification accuracy of 99.5%. These results demonstrate that the network can simultaneously provide geometric grasp information and semantic category information for workshop-oriented robotic manipulation.

The grasp-only configuration achieves an accuracy of 98.5%, which is 0.5 percentage points higher than the grasp accuracy of the multi-task model. This result indicates mild negative transfer between grasp detection and object classification. The classification branch does not necessarily improve grasp detection accuracy, but is retained because semantic category information is required for downstream sorting and placement.

The overall ablation experiments demonstrate that both GCSAFormer and SCRGM contribute to the final performance. The PoolFormer + GG baseline achieves grasp and classification accuracy results of 94.0% and 95.5%, respectively. Replacing the original grasping head with SCRGM increases these results to 96.0% and 99.0%, while replacing PoolFormer with GCSAFormer produces accuracy of 95.0% and 99.0%. The complete GCSAFormer + SCRGM configuration achieves the highest grasp accuracy of 98.0% and classification accuracy of 99.5%.

The component-level experiments further demonstrate that channel attention, channel shuffling, and spatial attention each contribute to GCSA. Channel attention improves feature discrimination along the channel dimension, channel shuffling facilitates interaction among different channel groups, and spatial attention emphasizes grasp-relevant regions. The complete combination provides the highest performance.

Comparisons with SE, ECA, CBAM, and Shuffle Attention indicate that GCSA provides a moderate but consistent improvement under the same experimental configuration. Therefore, the contribution of GCSA is interpreted as a task-oriented organization of established channel and spatial feature processing operations rather than as the introduction of an entirely new attention principle.

The SCRGM experiments show that the SRU and CRU both improve grasp prediction. The SRU primarily reorganizes spatially redundant responses, while the CRU refines correlated channel information. Their sequential combination produces the highest grasp accuracy, supporting the use of spatial and channel reconstruction in the grasping head. Because the reconstruction principles are related to ScConv, the task-specific contribution of SCRGM lies in its integration with multi-scale fused features, progressive upsampling, and dense grasp prediction.

The multi-scale feature fusion experiments demonstrate that combining features from different backbone stages progressively improves grasp performance. Deep features provide high-level semantic and contextual information, whereas shallow features preserve object boundaries, local contours, and fine geometric details. Together, their integration benefits the dense estimation of grasp center, orientation, and gripper opening.

The input resolution experiments show that increasing the image resolution from 224×224 to 300×300 improves both grasp detection and classification. Increasing the resolution beyond 300×300 produces only marginal performance gains; therefore, 300×300 is selected as the default resolution because it preserves sufficient spatial detail while avoiding an unnecessarily large input representation.

Finally, the physical robotic experiments demonstrate the feasibility of integrating MTGCSAFormer with an eye-in-hand RGB-D robotic system. The robot successfully completed 76 of 80 grasp attempts, corresponding to an overall success rate of 95.0%. Workshop tools and mechanical parts achieve a success rate of 97.5%, while additional daily objects achieve 92.5%.

The physical experiments also clarify the roles of RGB and depth information. MTGCSAFormer uses RGB images for feature extraction, classification, and planar grasp prediction. The aligned depth image is used only after network inference to recover the metric three-dimensional position of the predicted grasp point. Camera intrinsic parameters, eye-in-hand calibration, and the current end-effector pose are then used to transform the grasp point into the robot base coordinate system.

Overall, the experimental results demonstrate that MTGCSAFormer provides accurate planar grasp prediction and object classification for workshop-oriented robotic manipulation. The improvements arise from the combined effects of hierarchical feature refinement, multi-scale feature aggregation, and spatial/channel reconstruction. At the same time, the experiments reveal limitations related to multi-task competition, dataset diversity, depth uncertainty, calibration error, and transfer to unseen hardware and environments.

## 4. Conclusions

This study presents MTGCSAFormer, a multi-task perception framework for planar robotic grasp detection and object classification in workshop-oriented manipulation scenarios. The network uses a hierarchical GCSAFormer backbone to extract channel- and spatially-refined features, multi-scale feature fusion to combine spatial details and semantic information, and SCRGM to reconstruct spatial and channel responses prior to dense grasp prediction.

The GCSA module organizes channel recalibration, channel shuffle, and spatial refinement into a sequential feature processing structure. The component-level ablation experiments show that each operation contributes to the final grasp performance. Comparisons with representative attention mechanisms further indicate that the proposed organization is suitable for dense grasp prediction, although the individual attention operations are based on established feature processing principles.

SCRGM integrates SRU and CRU into the grasping head to refine the multi-scale fused representation. The experimental results show that SRU and CRU provide complementary improvements by addressing spatially repeated responses and correlated channel information, respectively. The reconstruction principles are related to ScConv, while the task-specific contribution of SCRGM lies in its integration with progressive feature recovery and dense prediction of grasp quality, orientation, and gripper opening.

On the Cornell Grasping Dataset, MTGCSAFormer achieves an accuracy of 99.5% under both image-wise and object-wise partitioning protocols. To evaluate the predicted grasp representation using the rectangle-based Cornell criterion, the predicted grasp center, orientation, and gripper opening are combined with a fixed 30-pixel short side to reconstruct an oriented evaluation rectangle. The standard intersection-over-union definition is used, and the sensitivity analysis indicates that the evaluation remains relatively stable around the selected short-side value.

On GTPWD, the complete multi-task model achieves 98.0% grasp detection accuracy and 99.5% object classification accuracy. The grasp-only model achieves 98.5%, revealing mild negative transfer between the two tasks. The multi-task architecture is nevertheless retained because the target robotic sorting scenario requires both grasp geometry and semantic category information.

The overall ablation results show that the PoolFormer + GG baseline achieves 94.0% grasp accuracy and 95.5% classification accuracy, while the complete GCSAFormer + SCRGM configuration achieves 98.0% and 99.5%, respectively. These results support the combined use of backbone-level channel–spatial feature refinement and grasp head-level spatial/channel reconstruction.

Physical experiments conducted on an AUBO-i3 manipulator achieve an overall grasp success rate of 95.0%. MTGCSAFormer uses RGB images for planar grasp prediction and object classification, while aligned depth information is used after inference for three-dimensional localization. Camera intrinsic parameters, eye-in-hand calibration, and the current robot pose are used to transform the predicted grasp point into an executable pose in the robot base coordinate system.

The current study remains limited by the scale and diversity of GTPWD, the use of a single robotic platform and camera configuration, the controlled single-target physical evaluation, and the focus on planar parallel-jaw grasping. In addition, multi-task competition, depth noise, calibration error, and gripper–object interaction can affect the final physical result.

Future work will expand the dataset to include more object categories, materials, viewpoints, backgrounds, and illumination conditions. Adaptive multi-task optimization, gradient conflict mitigation, uncertainty-aware depth processing, instance-level multi-object grasping, cross-camera and cross-robot evaluation, and six-degrees-of-freedom grasp prediction will also be investigated to improve the generalization and robustness of the system in more complex industrial environments.

## Figures and Tables

**Table 1:** Main characteristics of representative planar grasp detection datasets.

Dataset	Data Type	Images	Objects	Annotations	Modality
Cornell	Real	885	240	8019	RGB-D
Jacquard	Synthetic	54 K	11 K	1.1 M	RGB-D

**Table 2:** Partitioning of the General Tools and Parts in Workshop Dataset.

Subset	Number of Images	Percentage
Training	1728	72%
Validation	192	8%
Test	480	20%

**Table 3:** Annotation consistency assessment of the GTPWD.

Metric	Result
Mean grasp center deviation	3.2 pixels
Mean orientation deviation	$4.7^{\circ }$
Mean grasp rectangle IoU	0.86
Annotation agreement rate	94.5%

**Table 4:** Training implementation hyperparameters.

Parameter	Setting
Hardware	AMD R9-7945HX; NVIDIA RTX 4060 8 GB
Framework	PyTorch
Input resolution	300×300
Backbone	GCSAFormer-S24
Training batch size	4
Optimizer	AdamW
Initial learning rate	$1\times 10^{-3}$
Training epochs	100
Iterations per epoch	1000
Average inference time	7 ms per image (network forward pass)
Data augmentation	Rotation, translation, scaling, flipping, brightness and contrast adjustment

**Table 5:** Data partitioning protocols used for the Cornell Grasping Dataset.

Protocol	Partition Unit	Training/Test Ratio	Evaluation Purpose
Image-wise (IW)	Individual images	80:20	Pose and viewpoint variation
Object-wise (OW)	Object identities	80:20	Generalization to unseen objects

**Table 6:** Comparison of different grasp detection methods on the Cornell Grasping Dataset.

Method	Year	IW (%)	OW (%)	Inference Time (ms)
GG-CNN [18]	2019	73.0	69.0	19.0
SE-ResUNet [21]	2022	98.2	97.1	25.0
AFFGA-Net [48]	2022	99.09	98.64	15.0
CLRG [49]	2023	98.9	98.9	16.0
Efficient-Grasp [50]	2023	97.8	97.8	6.0
SISG-Net [6]	2023	98.9	98.9	18.5
MSG-ConvNet [5]	2023	98.3	98.6	7.0
UFGNet [25]	2025	98.4	–	29.0
LPGNet [26]	2026	98.31	97.84	14.2
MTGCSAFormer	–	**99.5**	**99.5**	**7.0**

**Table 7:** Sensitivity of grasp detection accuracy to the short-side length used for constructing the evaluation rectangle.

$h_{e}$ (Pixels)	IW Accuracy (%)	OW Accuracy (%)
20	99.1	99.0
25	99.3	99.2
30	**99.5**	**99.5**
35	99.4	99.3
40	99.2	99.1

**Table 8:** Comparison between grasp-only and multi-task configurations on GTPWD.

Configuration	Grasp Accuracy (%)	Classification Accuracy (%)
Grasp-only	**98.5**	–
Multi-task MTGCSAFormer	98.0	**99.5**

**Table 9:** Influence of the classification loss weight on multi-task performance.

$\lambda_{\mathrm{cls}}$	Grasp Accuracy (%)	Classification Accuracy (%)
0	**98.5**	–
0.01	98.4	98.6
0.05	98.2	99.2
0.10	98.0	**99.5**
0.20	97.7	99.5
0.50	97.2	99.5

**Table 10:** Ablation results of GCSAFormer and SCRGM on GTPWD.

Configuration	Grasp Accuracy (%)	Classification Accuracy (%)
PoolFormer + GG	94.0	95.5
PoolFormer + SCRGM	96.0	99.0
GCSAFormer + GG	95.0	99.0
GCSAFormer + SCRGM	**98.0**	**99.5**

**Table 11:** Component-level ablation results of GCSA on GTPWD.

CA	CS	SA	Grasp Accuracy (%)	Classification Accuracy (%)
–	–	–	96.0	99.0
✓	–	–	96.7	99.1
–	–	✓	96.5	99.1
✓	✓	–	97.2	99.3
✓	–	✓	97.5	99.3
✓	✓	✓	**98.0**	**99.5**

**Table 12:** Comparison of different attention mechanisms on GTPWD.

Attention Mechanism	Grasp Accuracy (%)	Classification Accuracy (%)
None	96.0	99.0
SE	96.5	99.1
ECA	96.7	99.1
CBAM	97.2	99.3
Shuffle Attention	97.5	99.3
GCSA	**98.0**	**99.5**

**Table 13:** Component-level ablation results of SCRGM on GTPWD.

SRU	CRU	Grasp Accuracy (%)	Classification Accuracy (%)
–	–	95.0	99.0
✓	–	96.2	99.1
–	✓	96.7	99.2
✓	✓	**98.0**	**99.5**

**Table 14:** Ablation results of multi-scale feature fusion on GTPWD.

Feature Levels	Grasp Accuracy (%)	Classification Accuracy (%)
$F_{4}$	96.6	99.0
$F_{3}+F_{4}$	97.1	99.2
$F_{2}+F_{3}+F_{4}$	97.6	99.4
$F_{1}+F_{2}+F_{3}+F_{4}$	**98.0**	**99.5**

**Table 15:** Influence of input resolution on the multi-task performance of MTGCSAFormer on GTPWD.

Input Resolution	Grasp Accuracy (%)	Classification Accuracy (%)
224×224	96.9	98.8
256×256	97.5	99.2
300×300	**98.0**	**99.5**
320×320	98.1	99.5
384×384	98.1	99.6

**Table 16:** Physical robotic grasping results.

Object Group	Attempts	Successful Grasps	Success Rate (%)
Workshop tools and parts	40	39	97.5
Daily objects	40	37	92.5
Overall	80	76	**95.0**

## Data Availability

The Cornell Grasping Dataset analyzed in this study is publicly available from its original source. The General Tools and Parts in Workshop Dataset (GTPWD) generated and analyzed during the current study is not publicly available at present because it is undergoing further organization and quality verification. The dataset may be made available by the corresponding author upon reasonable request, subject to institutional data sharing requirements.

## Abbreviations

The following abbreviations are used in this manuscript:

MTGCSAFormer	Multi-Task Global Channel–Spatial Attention Former
GCSA	Global Channel–Spatial Attention
SCRGM	Spatial and Channel Reconstruction Grasping Module
SRU	Spatial Reconstruction Unit
CRU	Channel Reconstruction Unit
MSFF	Multi-Scale Feature Fusion
GTPWD	General Tools and Parts in Workshop Dataset
GG	Original Grasping Head
GG-CNN	Generative Grasping Convolutional Neural Network
RGB	Red, Green, and Blue
RGB-D	Red, Green, and Blue with Depth
IW	Image-Wise Split
OW	Object-Wise Split
IoU	Intersection over Union
MSE	Mean Squared Error
MLP	Multi-Layer Perceptron
GAP	Global Average Pooling
CA	Channel Attention
CS	Channel Shuffle
SA	Spatial Attention
PnP	Perspective-*n*-Point

## References

[1] Tian H., Song K., Li S., Ma S., Xu J., Yan Y. (2023). Data-driven robotic visual grasping detection for unknown objects: A problem-oriented review. Expert Syst. Appl..

[2] Xie Z., Liang X., Roberto C. (2023). Learning-based robotic grasping: A review. Front. Robot. AI.

[3] Semeraro F., Griffiths A., Cangelosi A. (2023). Human–robot collaboration and machine learning: A systematic review of recent research. Robot. Comput.-Integr. Manuf..

[4] Du G., Wang K., Lian S., Zhao K. (2021). Vision-based robotic grasping from object localization, object pose estimation to grasp estimation for parallel grippers: A review. Artif. Intell. Rev..

[5] Duan S., Tian G., Wang Z., Liu S., Feng C. (2023). A Semantic Robotic Grasping Framework Based on Multi-Task Learning in Stacking Scenes. Eng. Appl. Artif. Intell..

[6] Yan Y., Tong L., Song K., Tian H., Man Y., Yang W. (2023). SISG-Net: Simultaneous instance segmentation and grasp detection for robot grasp in clutter. Adv. Eng. Inform..

[7] Zhong X., Chen Y., Luo J., Shi C., Hu H. (2024). A novel grasp detection algorithm with multi-target semantic segmentation for a robot to manipulate cluttered objects. Machines.

[8] Hu J., Shen L., Albanie S., Sun G., Wu E. Squeeze-and-Excitation Networks. Proceedings of the IEEE Conference on Computer Vision and Pattern Recognition.

[9] Wang Q., Wu B., Zhu P., Li P., Zuo W., Hu Q. ECA-Net: Efficient Channel Attention for Deep Convolutional Neural Networks. Proceedings of the IEEE/CVF Conference on Computer Vision and Pattern Recognition.

[10] Woo S., Park J., Lee J.Y., Kweon I.S. CBAM: Convolutional Block Attention Module. Proceedings of the European Conference on Computer Vision.

[11] Zhang Q.L., Yang Y.B. (2021). SA-Net: Shuffle Attention for Deep Convolutional Neural Networks. arXiv.

[12] Li J., Wen Y., He L. SCConv: Spatial and channel reconstruction convolution for feature redundancy. Proceedings of the IEEE/CVF Conference on Computer Vision and Pattern Recognition (CVPR).

[13] Fang H.S., Wang C., Gou M., Lu C. GraspNet-1Billion: A Large-Scale Benchmark for General Object Grasping. Proceedings of the IEEE/CVF Conference on Computer Vision and Pattern Recognition.

[14] Sundermeyer M., Mousavian A., Triebel R., Fox D. Contact-GraspNet: Efficient 6-DoF Grasp Generation in Cluttered Scenes. Proceedings of the 2021 IEEE International Conference on Robotics and Automation.

[15] Wang C., Fang H.S., Gou M., Fang H., Gao J., Lu C. Graspness Discovery in Clutters for Fast and Accurate Grasp Detection. Proceedings of the IEEE/CVF International Conference on Computer Vision.

[16] Fang H.S., Wang C., Fang H., Gou M., Liu J., Yan H., Liu W., Xie Y., Lu C. (2023). AnyGrasp: Robust and Efficient Grasp Perception in Spatial and Temporal Domains. IEEE Trans. Robot..

[17] Redmon J., Angelova A. (2014). Real-Time Grasp Detection Using Convolutional Neural Networks. arXiv.

[18] Morrison D., Corke P., Leitner J. (2018). Closing the loop for robotic grasping: A real-time, generative grasp synthesis approach. arXiv.

[19] Kumra S., Joshi S., Sahin F. Antipodal robotic grasping using generative residual convolutional neural network. Proceedings of the 2020 IEEE/RSJ International Conference on Intelligent Robots and Systems (IROS).

[20] Xu R., Chu F.J., Vela P.A. (2022). GKNet: Grasp keypoint network for grasp candidates detection. Int. J. Robot. Res..

[21] Yu S., Zhai D.H., Xia Y., Wu H., Liao J. (2022). SE-ResUNet: A Novel Robotic Grasp Detection Method. IEEE Robot. Autom. Lett..

[22] Wang H., Liu Z., Zhou L., Yin H., Ang M.H. (2022). PEGG-Net: Pixel-Wise Efficient Grasp Generation in Complex Scenes. arXiv.

[23] Zhai D.H., Yu S., Xia Y. (2024). FANet: Fast and Accurate Robotic Grasp Detection Based on Keypoints. IEEE Trans. Autom. Sci. Eng..

[24] Nie H., Zhao Z., Chen L., Lu Z., Li Z., Yang J. (2024). Smaller and Faster Robotic Grasp Detection Model via Knowledge Distillation and Unequal Feature Encoding. IEEE Robot. Autom. Lett..

[25] Miao H., Xu X., Yang Y. (2025). Pixel-Level Grasp Pose Detection Based on the Fusion of Convolution and Self-Attention. Manuf. Autom..

[26] Song L., Li C., Fu X., Yi Y. (2026). LPGNet: A Lightweight Pixel-Level Grasp Detection Network Based on Multi-Scale Features. Mech. Eng. Technol..

[27] Wang S., Zhou Z., Kan Z. (2022). When Transformer Meets Robotic Grasping: Exploits Context for Efficient Grasp Detection. IEEE Robot. Autom. Lett..

[28] Yu W., Luo M., Zhou P., Si C., Zhou Y., Wang X. MetaFormer is actually what you need for vision. Proceedings of the IEEE/CVF Conference on Computer Vision and Pattern Recognition (CVPR).

[29] Ma H., Shi M., Gao B., Huang D. Generalizing 6-DoF Grasp Detection via Domain Prior Knowledge. Proceedings of the IEEE/CVF Conference on Computer Vision and Pattern Recognition.

[30] Jauhri S., Lunawat I., Chalvatzaki G. Learning Any-View 6DoF Robotic Grasping in Cluttered Scenes via Neural Surface Rendering. Proceedings of the Robotics: Science and Systems.

[31] Iwase S., Irshad M.Z., Liu K., Guizilini V., Lee R., Ikeda T., Amma A., Nishiwaki K., Kitani K., Ambrus R. ZeroGrasp: Zero-Shot Shape Reconstruction Enabled Robotic Grasping. Proceedings of the IEEE/CVF Conference on Computer Vision and Pattern Recognition.

[32] Vandenhende S., Georgoulis S., Van Gansbeke W., Proesmans M., Dai D., Van Gool L. (2022). Multi-Task Learning for Dense Prediction Tasks: A Survey. IEEE Trans. Pattern Anal. Mach. Intell..

[33] Yang Y., Yu H., Lou X., Liu Y., Choi C. (2024). Attribute-Based Robotic Grasping with Data-Efficient Adaptation. IEEE Trans. Robot..

[34] Ainetter S., Fraundorfer F. End-to-End Trainable Deep Neural Network for Robotic Grasp Detection and Semantic Segmentation from RGB. Proceedings of the 2021 IEEE International Conference on Robotics and Automation (ICRA).

[35] Kamel M.S.A., Naish M.D. Mask-Grasp R-CNN: Simultaneous Instance Segmentation and Robotic Grasp Detection. Proceedings of the 2021 IEEE EMBS International Conference on Biomedical and Health Informatics (BHI).

[36] Zuo G., Tong J., Liu H., Chen W., Li J. (2021). Graph-Based Visual Manipulation Relationship Reasoning Network for Robotic Grasping. Front. Neurorobot..

[37] Zhang H., Lan X., Zhou X., Tian Z., Zhang Y., Zheng N. Visual Manipulation Relationship Network for Autonomous Robotics. Proceedings of the 2018 IEEE-RAS 18th International Conference on Humanoid Robots (Humanoids).

[38] Park D., Seo Y., Shin D., Choi J., Chun S.Y. A Single Multi-Task Deep Neural Network with Post-Processing for Object Detection with Reasoning and Robotic Grasp Detection. Proceedings of the 2020 IEEE International Conference on Robotics and Automation (ICRA).

[39] Vuong A.D., Vu M.N., Huang B., Nguyen N., Le H., Vo T. Language-driven grasp detection. Proceedings of the IEEE/CVF Conference on Computer Vision and Pattern Recognition (CVPR).

[40] Li T., Yan Y., Yu C., An J., Wang Y., Chen G. (2024). A comprehensive review of robot intelligent grasping based on tactile perception. Robot. Comput.-Integr. Manuf..

[41] Li J., Dong S., Adelson E.H. (2018). Slip Detection with Combined Tactile and Visual Information. arXiv.

[42] Mao Z., Wang J., Zhang J., Ohgi J., Zheng Y., Peng Y., Zhao L., Su Q., Huang W., Xu B. (2026). Fine-Tuned Multimodal Large Language Model for Autonomous State Cognition System of Shape-Recognition 6-Bar Tensegrity Integrated with Flexible Sensors. Microsyst. Nanoeng..

[43] Yu T., Kumar S., Gupta A., Levine S., Hausman K., Finn C. Gradient Surgery for Multi-Task Learning. Proceedings of the Advances in Neural Information Processing Systems.

[44] Tsai R.Y., Lenz R.K. (1989). A New Technique for Fully Autonomous and Efficient 3D Robotics Hand/Eye Calibration. IEEE Trans. Robot. Autom..

[45] Daniilidis K. (1999). Hand-Eye Calibration Using Dual Quaternions. Int. J. Robot. Res..

[46] Lenz I., Lee H., Saxena A. (2015). Deep learning for detecting robotic grasps. Int. J. Robot. Res..

[47] Depierre A., Dellandrea E., Chen L. Jacquard: A Large Scale Dataset for Robotic Grasp Detection. Proceedings of the 2018 IEEE/RSJ International Conference on Intelligent Robots and Systems (IROS).

[48] Wang D., Liu C., Chang F., Li N., Li G. (2022). High-performance pixel-level grasp detection based on adaptive grasping and grasp-aware network. IEEE Trans. Ind. Electron..

[49] Liu J., Xie J., Huang S., Wang C., Zhou F. (2023). Continual learning for robotic grasping detection with knowledge transferring. IEEE Trans. Ind. Electron..

[50] Cao H., Chen G., Li Z., Feng Q., Lin J., Knoll A. (2023). Efficient grasp detection network with Gaussian-based grasp representation for robotic manipulation. IEEE/ASME Trans. Mechatron..

